# Randomized phase II study of preoperative afatinib in untreated head and neck cancers: predictive and pharmacodynamic biomarkers of activity

**DOI:** 10.1038/s41598-023-49887-4

**Published:** 2023-12-18

**Authors:** Grégoire Marret, Stéphane Temam, Maud Kamal, Caroline Even, Jean-Pierre Delord, Caroline Hoffmann, Gilles Dolivet, Olivier Malard, Jérôme Fayette, Olivier Capitain, Sébastien Vergez, Lionel Geoffrois, Frédéric Rolland, Philippe Zrounba, Laurent Laccourreye, Esma Saada-Bouzid, Nicolas Aide, Valérie Bénavent, Jerzy Klijianenko, Constance Lamy, Elodie Girard, Sophie Vacher, Julien Masliah-Planchon, Leanne de Koning, Vincent Puard, Edith Borcoman, Marta Jimenez, Ivan Bièche, Jocelyn Gal, Christophe Le Tourneau

**Affiliations:** 1 Department of Drug Development and Innovation (D3i), Institut Curie, 26 Rue d’Ulm, 75005 Paris, France; 2 Department of Head and Neck Surgery, Gustave Roussy, Villejuif, France; 3 Head and Neck Oncology Department, Gustave Roussy, Villejuif, France; 4 Department of Medical Oncology, Centre Claudius Régaud, Toulouse, France; 5 Department of Head and Neck Surgery, Institut Curie, Paris, France; 6 Department of Head and Neck Surgery, Institut de Cancérologie de Lorraine, Nancy, France; 7 Department of Head and Neck Surgery, Centre Hospitalier Universitaire, Nantes, France; 8 Department of Medical Oncology, Centre Léon Bérard, Lyon, France; 9 Department of Medical Oncology, Centre Paul Papin, Angers, France; 10 Department of Head and Neck Surgery, Institut Claudius Regaud, Toulouse, France; 11 Department of Medical Oncology, Institut de Cancérologie de Lorraine, Nancy, France; 12 Department of Medical Oncology, Centre René Gauducheau, Nantes, France; 13 Department of Head and Neck Surgery, Centre Léon Bérard, Lyon, France; 14 Department of Head and Neck Surgery, Centre Hospitalier Universitaire, Angers, France; 15 Department of Medical Oncology, Centre Antoine Lacassagne, Nice, France; 16 Department of Nuclear Medicine, Centre François Baclesse, Caen, France; 17 UNICANCER, Paris, France; 18 Department of Pathology, Institut Curie, Paris, France; 19 Bioinformatics Core Facility, INSERM U900, Mines Paris Tech, Institut Curie, Paris, France; 20 Genetics Department, Institut Curie, Paris, France; 21 Department of Translational Research, Institut Curie, PSL Research University, Paris, France; 22 Department of Biostatistics, Centre Antoine Lacassagne, Nice, France; 23 INSERM U900, Institut Curie, Paris-Saclay University, Paris, France

**Keywords:** Head and neck cancer, Predictive markers, Next-generation sequencing, Proteomics, Pharmacodynamics

## Abstract

There is no strong and reliable predictive biomarker in head and neck squamous cell carcinoma (HNSCC) for EGFR inhibitors. We aimed to identify predictive and pharmacodynamic biomarkers of efficacy of afatinib, a pan-HER tyrosine kinase inhibitor, in a window-of-opportunity trial (NCT01415674). Multi-omics analyses were carried out on pre-treatment biopsy and surgical specimen for biological assessment of afatinib activity. Sixty-one treatment-naïve and operable HNSCC patients were randomised to afatinib 40 mg/day for 21–28 days versus no treatment. Afatinib produced a high rate of metabolic response. Responders had a higher expression of pERK1/2 (*P* = 0.02) and lower expressions of pHER4 (*P* = 0.03) and pRB1 (*P* = 0.002) in pre-treatment biopsy compared to non-responders. At the cellular level, responders displayed an enrichment of tumor-infiltrating B cells under afatinib (*P* = 0.02). At the molecular level, NF-kappa B signaling was over-represented among upregulated genes in non-responders (*P* < 0.001; FDR = 0.01). Although exploratory, phosphoproteomics-based biomarkers deserve further investigations as predictors of afatinib efficacy.

## Introduction

Head and neck squamous cell carcinoma (HNSCC) is the seventh most common cancer worldwide^1^. Early-stage disease can be successfully treated with a single-modality treatment (surgery or radiotherapy), whereas locally advanced disease usually uses multimodality treatments that involve surgery and (chemo)radiotherapy. Around 50% of locally advanced HNSCC recur after primary treatment, at locoregional and/or distant metastatic levels^2^. Patients who are not amenable to local treatments have a dismal prognosis (i.e., 6 to 9 months in the absence of treatment)^3^.

Increased or sustained activation of the epidermal growth factor receptor’s (EGFR) signaling can promote genesis and progression of tumors by providing sustained signals for cell proliferation, anti-apoptotic signaling, angiogenesis, and metastasis^4–6^. Overexpression of EGFR is a common characteristic of HNSCC and is associated with poor outcomes^7,8^.

Cetuximab, a monoclonal antibody (mAb) targeting EGFR, is to date the only targeted therapy that demonstrated an overall survival (OS) benefit in HNSCC patients, both in the recurrent and in the locally advanced settings, yet without molecular selection^9,10^. Lack of durable efficacy due to drug resistance remains a major challenge^11^. Unlike lung cancers, in which sensitizing *EGFR* mutations predict sensitivity to tyrosine kinase inhibitors (TKIs)^12,13^, no robust predictive biomarker for cetuximab efficacy has been identified in HNSCC^14,15^. Various innate and/or acquired resistance mechanisms have been reported^16–18^, including activation of other human epidermal growth factor receptors (HERs).

Pan-HER TKIs target all the dimers formed by HER family^19–22^ and have the potential to overcome cetuximab resistance caused by cross-talk between the EGFR and other HERs^23^. Afatinib, a second-generation pan-HER TKI, is a highly selective, potent, and irreversible inhibitor of EGFR, HER2, and HER4 kinases. It also prevents the trans-phosphorylation of HER3^20,22^. First-line afatinib demonstrated significant improvements in progression-free survival (PFS) and OS for patients with *EGFR*-mutated non-small cell lung cancers (NSCLC)^24,25^. In unselected HNSCC patients, afatinib modestly improved PFS as compared to methotrexate in second-line recurrent and/or metastatic setting^26^. Biomarkers analyses showed that PFS was improved in p16-negative, EGFR-amplified, HER3-low, and PTEN-high in this latter patient population^27^. In a preoperative trial led by the EORTC, afatinib given for two weeks to treatment-naïve, operable HNSCC patients was safe and produced a 70% rate of metabolic response^28^. None of the aforementioned biomarkers was predictive of response to treatment in this study.

The GEP11 PREDICTOR randomized trial aimed at identifying predictive and pharmacodynamic biomarkers of efficacy of afatinib. The trial also investigated the efficacy and safety of preoperative afatinib in untreated, non-metastatic HNSCC patients.

## Results

### Patients and treatment

**Figure 1:**
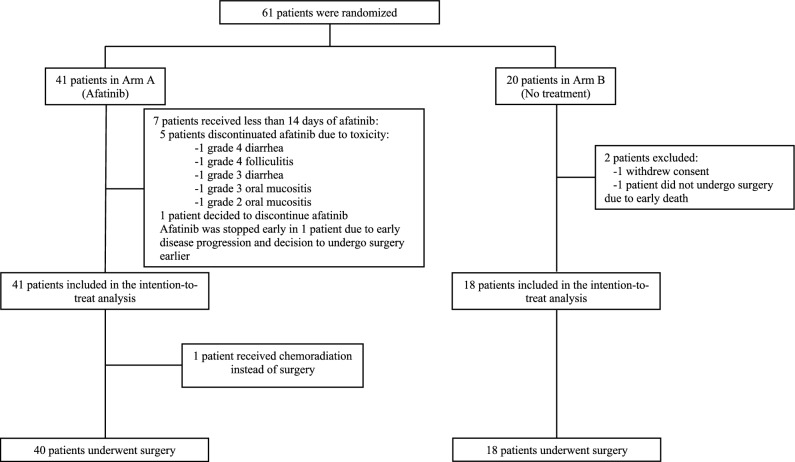
Consort diagram.

Sixty-one patients were included between January 2012 and July 2015 at seven French cancer centers or university hospitals and were randomized to either afatinib (*N* = 41; Arm A) or no treatment (*N* = 20; Arm B; Fig. 1). All patients in Arm A were included in the intention-to-treat population, whereas in Arm B, two out of 20 patients (10%) were excluded (one consent withdrawal and one early death before the scheduled surgery). In Arm A, one patient deemed inoperable following 14 days of afatinib received chemoradiation instead of surgery. Patient characteristics are described in Table 1.

**Table 1:** Patient characteristics.

	Arm A (*N* = 41)	Arm B (*N* = 18)	Total (*N* = 59)
Age, years			
Median	58.5	60.2	59.5
Range	44–74	46–76	44–76
Sex, n (%)			
Male	32 (78%)	15 (83%)	47 (80%)
Female	9 (22%)	3 (17%)	12 (20%)
ECOG status, n (%)			
Unknown	1 (2%)	0	1 (2%)
0	27 (66%)	13 (75%)	40 (68%)
1	13 (32%)	5 (25%)	18 (31%)
Smoker, n (%)			
Current	24 (59%)	7 (39%)	31 (53%)
Former	12 (29%)	8 (44%)	20 (34%)
Never	5 (12%)	3 (17%)	8 (14%)
Alcohol consumption, n (%)			
Current	22 (54%)	10 (56%)	32 (54%)
Former	13 (32%)	4 (22%)	17 (29%)
Never	6 (15%)	4 (22%)	10 (17%)
Primary tumor site, n (%)			
Oral cavity	26 (63%)	12 (67%)	38 (64%)
Larynx	0	1 (6%)	1 (2%)
Oropharynx	11 (27%)	3 (17%)	14 (24%)
Hypopharynx	4 (10%)	2 (11%)	6 (10%)
Histologic grade, n (%)			
Unknown	1 (2%)	0	1 (2%)
Well differentiated	21 (51%)	11 (61%)	32 (54%)
Moderately differentiated	12 (29%)	5 (28%)	17 (29%)
Poorly differentiated	7 (17%)	2 (11%)	9 (15%)
HPV status, n (%)			
Unknown	6 (15%)	0	6 (10%)
HPV negative	30 (73%)	16 (89%)	46 (78%)
HPV positive	5 (12%)	2 (11%)	7 (12%)
Pretreatment T-stage^a^, n (%)			
T1	1 (2%)	0	1 (2%)
T2	15 (37%)	4 (22%)	19 (32%)
T3	5 (12%)	4 (22%)	9 (15%)
T4	20 (49%)	10 (56%)	30 (51%)
Pretreatment N-stage^a^, n (%)			
N0	17 (41%)	12 (67%)	29 (49%)
N1	5 (12%)	2 (11%)	7 (12%)
N2	19 (46%)	4 (22%)	23 (39%)

^a^American Joint Committee on Cancer, 7th edition staging.

### Safety and tolerability

The median duration of afatinib treatment was 16 days (range, 3–30 days). Afatinib-related adverse events (AEs) are summarized in the Supplementary Table 1. Five patients discontinued afatinib within 14 days due to afatinib-related AEs, including one grade 4 diarrhea, one grade 4 folliculitis, one grade 3 oral mucositis, one grade 3 diarrhea, and one grade 2 oral mucositis. Three patients were still experiencing afatinib-related AEs three months after surgery, including one grade 2 anorexia, one grade 2 folliculitis, one grade 2 diarrhea, and one grade 1 folliculitis. Mean time between enrolment and surgery was similar in Arm A and Arm B (23.4 *versus* 20.5 days, *P* = 0.2).

### Efficacy outcomes

Median follow-up was 82.7 months (interquartile range [IQR], 69.8–90.6 months) in Arm A, and 76.6 months (IQR, 70.8–91.5 months) in Arm B. In Arm A, three out of the 41 patients (7.3%; 95% CI, 1.5–19.9) achieved a partial response according to Response Evaluation Criteria in Solid Tumors version 1.1 (RECIST1.1) *versus* no patients in Arm B (*P* = 0.03; Table 2). A median reduction of the sum of the target lesions of 4.8 mm was observed between baseline and preoperative imaging in Arm A (95% CI, 2.9–6.8; *P* < 0.001), and a median of 1.9 mm increase in Arm B (95% CI, 0–3.8; *P* = 0.05). In Arm A, 24 out of the 41 patients (58.5%; 95% CI, 42.1–73.7) achieved a partial metabolic response *versus* no patients in Arm B (*P* < 0.01).

**Table 2:** Efficacy of preoperative afatinib *versus* no treatment on CT scan/MRI according to RECIST1.1 and FDG-PET scan according to PERCIST.

n (%)	CT scan/MRI		FDG-PET scan	
	Arm A (*N* = 41)	Arm B (*N* = 18)	Arm A (*N* = 41)	Arm B (*N* = 18)
PR	3 (7.3%)	0	24 (58.5%)	0
SD	34 (82.9%)	14 (77.7%)	14 (34.1%)	13 (72.2%)
PD	0	3 (16.6%)	1 (2.4%)	3 (16.7%)
Non-evaluable	4 (9.8%)	1 (5.7%)	2 (5.0%)	2 (11.1%)
*P*-value	**0.03**		** < 0.01**	

*SD* stable disease, *PD* progressive disease, *PR* partial response.

Significant values are in bold.

Positive lymph nodes were diagnosed on resected tumors in 21 out of 41 patients (51%) in Arm A and in 11 out of 18 patients (61%) in Arm B. Among patients with positive lymph nodes, 12 (29%) displayed an extracapsular spread in Arm A *versus* four (22%) in Arm B. Statistical significance was not reached for a difference in the number of patients with positive lymph nodes or extracapsular spreads between the two arms (*P* = 0.5 and *P* = 0.8, respectively). In addition, microscopic residual disease assessed in the primary tumor was similar in Arm A and Arm B (mean [standard deviation] size, 3.3 [1.5] *versus* 4.3 [2.2] cm; *P* = 0.09).

### Exploratory DNA sequencing (DNAseq) analyses

DNAseq was performed on baseline tumor biopsies from 56 patients, including 37 patients in Arm A and 19 patients in Arm B. Among the 37 patients in Arm A, 35 were evaluable for FDG-positron emission tomography (FDG-PET) response (22 responders and 13 non-responders; Supplementary Fig. 1).

### Genomic alterations and signaling pathways

**Figure 2:**
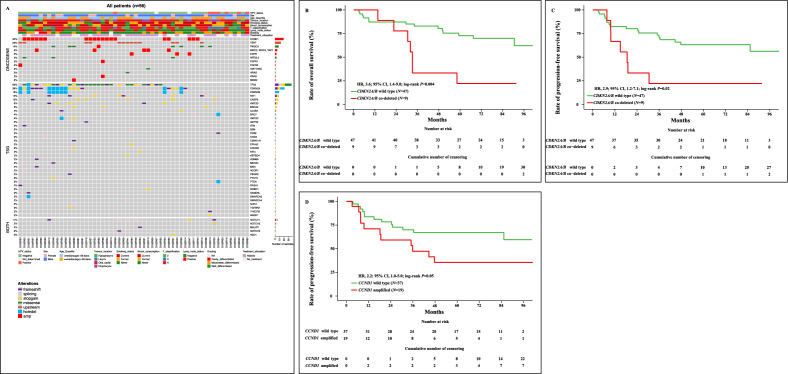
Overview of gene alterations in untreated, operable, non-metastatic HNSCC patients (*N* = 56) (**A**). Prognostic value of *CDKN2A/B* co-deletion on overall survival (**B**), and progression-free survival (**C**). Prognostic value of *CCND1* amplification on progression-free survival (**D**).

Among the 56 patients with contributive DNAseq, 49 (88%) had at least one molecular alteration. Thirty-five patients (62%) had an alteration in *TP53*, 21 (38%) in *CDKN2A*, 19 (34%) in *CCND1*, eight (14%) in *PIK3CA* and *FAT1*, and three (5%) in *EGFR*. Pathogenic promoter mutations in *TERT* were found in 13 patients (23%). Inactivating mutations in *CDKN2A* were found in 12 patients (22%) while *CDKN2A* and *CDKN2B* were co-deleted in nine patients (16%; Fig. 2A).

Genomic alterations were observed in the genome integrity signaling pathway in 35 patients (62%). Thirty-three patients (59%) had an alteration in the cell cycle pathway, 15 (27%) in the chromatin organization pathway, 13 (23%) in the senescence pathway, 12 (21%) in the apoptosis and the hippo signaling pathways, nine (16%) in the RTK/RAS and the Wnt signaling pathways, and seven (12%) in the PI3K and transcription factor regulator pathways (Supplementary Fig. 2). Proportions of genomic alterations and altered molecular pathways were similar in both treatment arms (Supplementary Fig. 3A, B).

### Predictive biomarkers of metabolic response to afatinib

Among the 35 patients evaluable for FDG-PET response in Arm A, none of the genomic alterations nor altered molecular pathways were associated with the metabolic response (Supplementary Table 2).

### Prognostic biomarkers

In post-hoc analyses, the prognostic value of genomic alterations and altered signaling pathways was assessed among the 56 patients with contributive DNAseq on baseline tumor (Supplementary Table 3). In univariate analysis, *CDKN2A/B* codeletion was associated with a shorter OS, with an HR of 3.6 (95% CI, 1.4–9.0; Fig. 2B). *CDKN2A/B* codeletion and *CCND1* amplification were also associated with a shorter PFS (HR, 2.9; 95% CI, 1.2–7.1; and HR, 2.2; 95% CI, 1.0–5.0, respectively; Fig. 2C, D). Among signaling pathways, alterations in the encompassing cell cycle pathway were associated with a shorter OS (HR, 3.8; 95% CI, 1.3–11.2) and PFS (HR, 2.7; 95% CI, 1.1–6.8; Supplementary Fig. 4A, B). In contrast with *CDKN2A/B* codeletion, *CDKN2A* mutation had no prognostic value on OS (HR, 1.4; 95% CI, 0.4–5.0) and PFS (HR, 0.8; 95% CI, 0.3–2.2; Supplementary Fig. 5A, B). *CCND1* amplification and *CDKN2A/B* codeletion had no prognostic value in multivariate analysis on OS and PFS (Supplementary Table 4).

We next focused on the top 10% of patients with the highest tumor mutational burden (TMB) scores corresponding to a TMB > 15.5 mut/Mb. There was no difference in OS between patients with high TMB (> 15.5 mut/Mb) and low TMB (≤ 15.5 mut/Mb), with an HR of 0.9 (95% CI, 0.22–4.0; Supplementary Fig. 6).

### Exploratory RNA sequencing (RNAseq) analyses

RNAseq was performed on baseline tumor biopsies from 53 patients, including 35 patients in Arm A and 18 patients in Arm B. Among the 35 patients in Arm A, 34 were evaluable for FDG-PET response (19 responders and 15 non-responders). Among them, 26 patients (15 responders and 11 non-responders) had both pre- and post-afatinib tumor samples with available gene expression data analysis (Supplementary Fig. 1).

### Cellular level characterization of pharmacodynamic biomarkers

**Figure 3:**
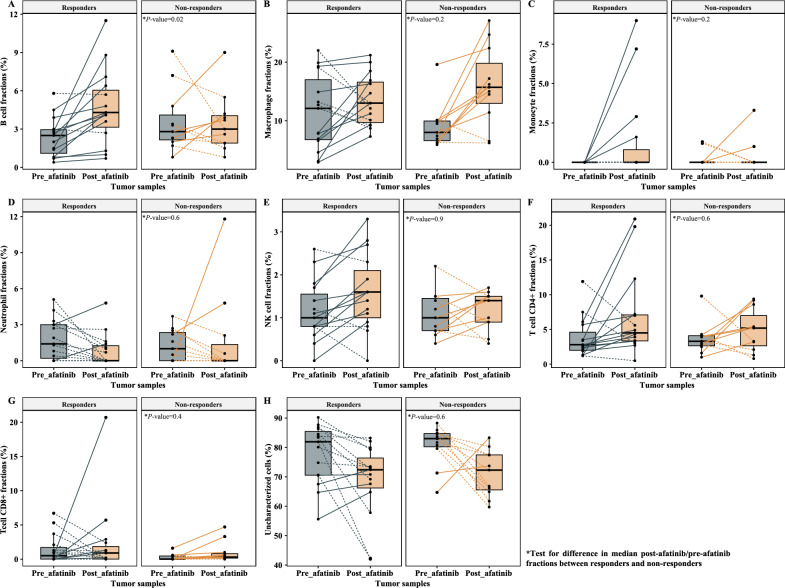
Tumor microenvironment cell fraction changes between pre- and post-afatinib tumor biopsies in responder (*N* = 14) and non-responder patients (*N* = 11) according to the metabolic response on FDG-PET scan by PERCIST. Tumor microenvironment cells included B cells (**A**), macrophages (**B**), monocytes (**C**), neutrophils (**D**), NK cells (**E**), T cells CD4+ (**F**) and T cells CD8+ (**G**). Uncharacterized cells, which were representative of cells outside immune subtypes (including tumor cells), were also reported (**H**).

We quantified the numbers and the types of tumor microenvironment (TME) cells among the 26 patients evaluable for FDG-PET response in Arm A with contributive paired pre- and post-afatinib RNAseq. We applied a deconvolution method on bulk gene expression data, and reported the TME cell fraction changes between pre- and post-afatinib tumor biopsies according to afatinib metabolic response on FDG-PET scan (Fig. 3). We further computed ratios of post-afatinib/pre-afatinib fractions (or differences in case of null denominator) for each type of TME cells, in responders (*N* = 15) and non-responders (*N* = 11; Table 3). We showed higher ratios for B cells in responder than in non-responder patients [median (range) ratio, 1.8 (0.9–5.9) versus 0.8 (0.3–5.3); *P* = 0.02]. In contrast, macrophages enrichment was numerically higher in non-responders compared to responders, albeit not statistically significant [median (range) ratio, 1.6 (0.7–3.3) versus 1.1 (0.6–5.4); *P* = 0.2].

**Table 3:** Variation of fraction of tumor-infiltrating immune cells under afatinib according to metabolic response on FDG-PET scan by PERCIST.

Immune cells	Non-responders (*N* = 11)				Responders (*N* = 15)				Ratio of median	*P*-value
	Median^¶^	Min	Max		Median^¶^	Min	Max			
B cells	0.8	0.3	5.3		1.8	0.9	5.9		2.1	**0.02**
Macrophages	1.6	0.7	3.3		1.1	0.6	5.4		0.7	0.2 (NS)
Monocytes	0	−0.01	0.03		0	0	0.09		–	0.2 (NS)
Neutrophils	−0.005	−0.03	0.1		−0.007	−0.05	0.02		1.4	0.6 (NS)
NK cells	1.4	0.3	2.5		1.3	0	3		0.9	0.9 (NS)
T cells CD4+	1.4	0.2	5.8		1.4	0.4	9.5		1.1	0.6 (NS)
T cells CD8+	0.003	−0.004	0.03		0	−0.05	0.2		0	0.4 (NS)
Uncharacterized cells^$^	0.9	0.7	1.3		0.9	0.5	1.2		1.1	0.6 (NS)

*NS* not significant.

^$^Uncharacterized cells are representative of cells outside immune subtypes, including tumor cells.

^¶^Median post-afatinib/pre-afatinib fractions were computed in B cells, macrophages, NK cells, T cells CD4+ and CD8+, and uncharacterized cells; median immune cell fractions derived by subtracting the post-afatinib cell fraction from the pre-afatinib cell fraction were computed in monocytes, neutrophils, and T cells CD8+.

Significant values are in bold.

### Molecular level characterization of pharmacodynamic biomarkers

**Figure 4:**
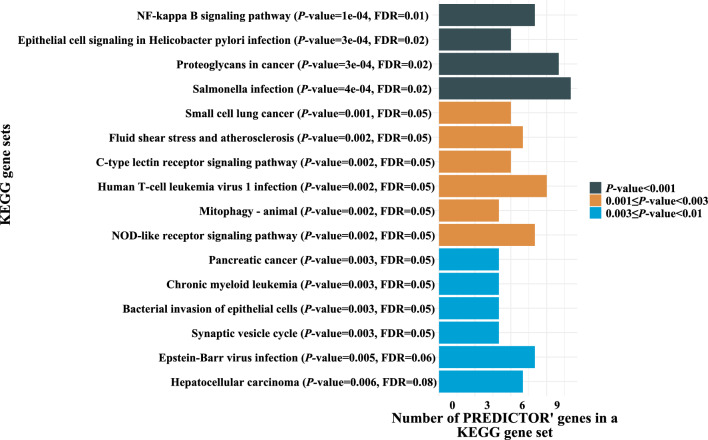
Gene sets which were found to be over-represented among up-regulated genes (*N* = 267) in non-responder patients, as determined by Over Representation Analysis (ORA). *KEGG* Kyoto Encyclopedia of Genes datasets, *FDR* false discovery rate.

Among the 26 aforementioned patients in Arm A, 600 genes were differentially expressed (267 and 333 upregulated in non-responders and responders, respectively), including the immune markers *ICAM1*, *MEF2C*, *P2RX5*, *HPGD*, and *ZBTB16* (Supplementary Table 5). We identified 16 gene sets **(**Fig. 4**)** over-represented among upregulated genes in non-responders, including the NF-kappa B signaling [*P* < 0.001; false discovery rate (FDR) = 0.01; Supplementary Fig. 7]. There was no evidence of enrichment among upregulated genes in responder patients.

### Prognostic biomarkers

Among the 53 patients with contributive RNAseq, post-hoc unsupervised analysis on bulk gene expression data (representing 13,232 transcripts) identified two clusters of patients (Cluster 1, *N* = 27; Cluster 2, *N* = 26). In univariate analysis, there was no difference in OS and PFS between these two clusters, with HRs of 0.5 (95% CI, 0.2–1.3) and 0.5 (95% CI, 0.2–1.2), respectively (Supplementary Fig. 8A, B). Clinical and histological characteristics of each cluster are presented in the Supplementary Table 6.

### Exploratory reverse phase protein arrays (RPPA) analyses

Protein extracts from baseline tumor biopsies were retrieved from 42 patients (26 patients in Arm A and 16 patients in Arm B) and were evaluated by RPPA methods by using a panel of 77 antibodies. Among the 26 patients in Arm A, 25 were evaluable for FDG-PET response (14 responders and 11 non-responders; Supplementary Fig. 1).

### Predictive biomarkers of metabolic response to afatinib

**Figure 5:**
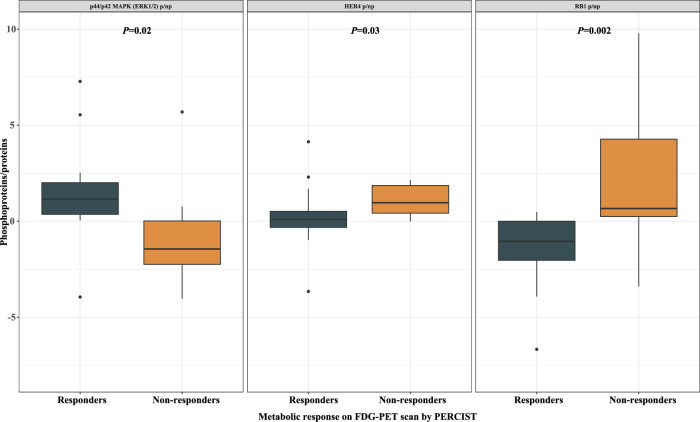
Correlation between phosphorylation levels of p44/p42 MAPK (ERK1/2), HER4, and RB1 and metabolic response on FDG-PET scan according to PERCIST (responders, *N* = 14; non-responders, *N* = 11). *p/np* phosphoproteins/proteins.

In the 25 patients evaluable for FDG-PET response in Arm A, we analyzed the relative expressions of phosphoproteins/proteins in pre-afatinib tumor biopsies according to metabolic response (Supplementary Fig. 9). We showed higher phosphorylation levels of p44/p42 MAPK (ERK1/2) in responders than in non-responders [median (range) ratio, 1.2 (−40.5 to 7.3) versus −1.4 (−4.0 to 5.7); *P* = 0.02] and lower phosphorylation levels of HER4 [median (range) ratio, 0.09 (−3.7 to 4.1) versus 1.0 (−0.002 to 122.0); *P* = 0.03] and RB1 [median (range) ratio, −1.0 (−14.7 to 0.5) versus 0.7 (−3.4 to 9.8); *P* = 0.002; Fig. 5].

### Prognostic biomarkers

Among the 42 patients with contributive RPPA, post-hoc unsupervised clustering on protein expression data identified two clusters of patients that did not significantly correlate with OS (HR, 1.7; 95% CI 0.6–4.6) or PFS (HR, 1.3; 95% CI, 0.5–3.4) in univariate analysis (data not shown).

## Discussion

Short course of afatinib in untreated, operable, non-metastatic HNSCC patients induced a high rate of partial metabolic responses and partial responses according to RECIST1.1 without delaying surgery. Baseline phosphorylation levels of RB1, ERK1/2, and HER4 correlated with metabolic responses to afatinib. At the cellular level, we showed a significant enrichment of tumor-infiltrating B cells under afatinib in responders, while at the molecular level, the NF-kappa B signaling was found to be enriched among upregulated genes in non-responders. In post-hoc analyses, we reported the negative prognostic values of *CCND1* amplification and *CDKN2A/B* codeletion although these results were not confirmed in multivariate analyses.

Seven percent of the patients achieved a partial response according to RECIST1.1, and 59% achieved a partial metabolic response as compared with 22% and 70% in a similar study run by the EORTC^28^. The lower response rates observed in our trial might be explained (1) by the higher proportion of patients with advanced disease at diagnosis (51% *versus* 33% of T4 stage tumors) and (2) by a difference in metabolic tumor response assessments, since in the EORTC study the last dose of afatinib was given strictly two hours before the FDG-PET scan before surgery.

Safety and tolerability were as expected regarding the toxicity profile of afatinib. Up to 12% of patients in Arm A stopped afatinib early because of toxicity, which might impact the results of translational analyses because of the lack of sufficient drug exposure. Importantly, all reported AEs were manageable and did not delay the planned definitive surgery, the latter representing a major concern with window-of-opportunity trials^29^.

In comparison with the TCGA cohort^30^, cell cycle regulatory genes were less frequently altered in our study, with lower rates of *CDKN2A* alteration (38% *versus* 50%) and *CDKN2A/B* codeletion (16% versus 27%). This might be explained by the fact that we applied a more stringent variant selection algorithm when taking into account copy number deletions. The negative prognostic impact of *CDKN2A* (that encodes p15) alteration is well established in HNSCC^31,32^, as opposed to *CDKN2B* (that encodes p16) homozygous deletion. We found that *CDKN2A/B* codeletion but not *CDKN2A* mutation alone correlated with poor survival outcomes, suggesting a key role of both p15 and p16 losses in determining the prognosis of *CDKN2A/B* co-deleted HNSCC.

We identified higher metabolic response to afatinib in patients with low pre-treatment HER4 activity. This result is not in line with what has been observed in preclinical model. A xenograft model of HNSCC identified a *HER4*-activating oncogenic mutation leading to increased HER4 phosphorylation, which suggested that it may activate other HERs as a heterodimer and predict sensitivity to afatinib^33^. These results highlight the controversial role of HER4 in cancers, which may act as a tumor suppressor protein or as an oncoprotein^34^. Our findings also support the hypothesis that constitutive activation of ERK1/2 (based on assessments of functional T202 and Y204 phosphosites) may serve as predictive biomarker of activity of afatinib. ERK1/2 induces the transcription and the translation of cyclin D1, thus promoting G1/S progression through cyclin D1-CDK4/6-mediated phosphorylation of RB1^35^. Unexpectedly, we reported lower relative expressions of phosphoproteins/proteins for RB1 in responder patients than in non-responder patients, suggesting other mechanisms of cell cycle activation besides CDK4/6-mediated phosphorylation of RB1.

At the cellular level, pharmacodynamic analyses on the TME elucidated B cells changes in response to afatinib which may rely on the (re)activation of a tumor-targeting immune response^36^. In contrast to B cells, macrophages enrichment upon afatinib was found to be higher in non-responders compared to responders, although the difference was not statistically significant (*P* = 0.2). A number of clinical studies in NSCLC have shown that the degree of infiltration of tumor-associated macrophages positively correlated with disease progression and resistance to EGFR-TKIs^37,38^. In addition, a syngeneic murine model of *EGFR*-mutant lung tumor recently highlighted the immunosuppressive effects of tumor-associated macrophages on T cells CD8^+^, thus impairing the efficacy of osimertinib (a third-generation EGFR-TKI)^39^. At the molecular level, Over Representation Analysis findings suggested mechanisms of resistance to afatinib associated with tumor invasion and metastasis through NF-kappa-B signaling enrichment^40,41^, a molecular feature of the 42-gene Chung’s high-risk signature^42^.

Our study has several limitations. First, mean tumor reduction was lower than anticipated and correlative biomarkers analyses were based instead on metabolic response, which was not a predefined primary endpoint. In addition, no adjustment for multi-test were carried out, thus rendering biomarkers analyses mainly exploratory. Second, the specificity of FDG-PET scan to predict afatinib efficacy is unknown. In some pre-clinical and clinical studies, however, metabolic responses to targeted therapies have been shown to correlate with residual tumor cellularity, tumor shrinkage, and improved time to progression^43–45^. Third, we did not confirm the biomarkers findings from the LUX-Head and Neck 1 trial^27^, which demonstrated a higher efficacy of afatinib in p16-negative, EGFR-amplified, HER3-low, and PTEN-high HNSCC patients. This can be due to the intrinsic nature of window-of-opportunity trial, in which patients are exposed to drugs during a short period of time, impairing the onset of potential acquired drug resistance. Fourth, recent data underscored the importance of tertiary lymphoid structure in TME and their interplay with B cells in eliciting antitumor responses^46,47^. We did not implement spatial information and functional orientation of tumor-infiltrating immune cells and related B cell phenotypes and functions. In this regard, our understanding of the immune contexture is limited. Fifth, our patient cohort was heterogeneous and encompassed various primary tumor sites, histologic grades, HPV status, and pre-treatment T- and N-stages, which may have impaired biomarkers discovery.

Overall, we found that short course of preoperative afatinib induced a high rate of metabolic response without delaying the planned definitive surgery in treatment-naïve HNSCC patients selected for primary curative surgery. Baseline expression of pHER4, pERK1/2 and pRB1 together with B cells enrichment and NF-kappa B signaling activation correlated with metabolic response to afatinib. Although exploratory, these phosphoproteomics-based biomarkers deserve further investigations as predictors of afatinib efficacy. More mechanistic studies are needed to reliably establish the link between B cells enrichment, NF-kappa B signaling activation and sensitivity to HER family blockers in HNSCC.

## Methods

### Study objectives and endpoints

The main objective consisted in identifying predictive biomarkers of efficacy by exploring correlation between baseline potential biomarkers and radiological and metabolic responses to afatinib. Secondary objectives were to identify potential pharmacodynamic biomarkers, to evaluate the efficacy and safety of afatinib and to assess the metabolic and pathologic responses. We also did post-hoc analyses to evaluate the prognostic significance of recurrent genomic alterations and TMB in HNSCC.

The primary endpoint was the biological assessment of afatinib activity by performing translational researches in pre-treatment biopsy and surgical specimen. Translational researches included DNAseq, RNAseq and high throughput protein analysis using RPPA methods.

For secondary endpoints, efficacy was defined as the tumor size reduction between baseline and before surgery. The tumor size was the sum of two target lesions following measurement rules on CT scan/MRI of the head and neck according to RECIST1.1^48^. Metabolic response was measured on FDG-PET scan according to Positron Emission Tomography Response Criteria in Solid Tumours (PERCIST)^49^. Patients with a partial metabolic response were considered as responders, whereas patients with a stable or a progressive metabolic disease were considered as non-responders. The toxicity was assessed according to the Common Terminology Criteria for Adverse Events (CTCAE) version 4.0. Pathologic response was assessed on resected lymph nodes by the presence or not of invasive tumor.

For post-hoc analyses, OS was defined as the time from randomization until death from any cause or last follow-up. PFS was defined as the time from randomization to disease progression or death from any cause or last follow-up.

### Study design and participants

The GEP11 PREDICTOR trial was an open-label, randomized, multicentric, controlled, phase II trial evaluating preoperative afatinib *versus* no treatment in patients with untreated, operable, non-metastatic T2-4 N0-2 (American Joint Committee on Cancer, 7th edition) HNSCC of the oral cavity, pharynx, and larynx. Randomization was stratified by primary tumor site (oropharynx *versus* non-oropharynx). All patients provided written informed consent before enrollment. The trial was approved by a national ethics committee. All methods were carried out in accordance with relevant guidelines and regulations. All experimental protocols were approved by UNICANCER.

### Treatment and procedures

Patients were randomized (2:1) to receive oral afatinib 40 mg/day for 21 to 28 days (Arm A) or no treatment (Arm B) before surgery. Surgery was not delayed as compared to standard of care. Tumor biopsies were collected during baseline endoscopy and surgery. FDG-PET scans were performed before the endoscopy, and at day 15 after treatment initiation. FDG-PET scans examinations were performed on the same system at baseline and before surgery, and assessed by central imaging review (FDG-PET unit of the François Baclesse Cancer Center) as per the EANM guidelines^50^. CT scan/MRI were performed before the endoscopy and after the end of treatment within one week prior to surgery.

### DNA sequencing

Samples were sequenced using a dedicated next-generation sequencing (NGS) panel targeting 571 genes (named “DRAGON”). Genes were classified regarding the literature and databases (cBioPortal and OncoKB^51,52^) in tumor suppressor genes (TSG), oncogenes, and genes considered as both an oncogene and a TSG. Genes were categorized according to the cellular pathway in which they were involved (Supplementary Table 7).

### DNAseq protocol

Indexed paired-end libraries of tumor DNA were performed using the Agilent Sureselect XT2 library prep kit. The kit supports sequencing targeted regions of the genome spanning 2.7 Mb. About 50 ng of input DNA were used to build the libraries according to manufacturer’s protocol. The pool was finally sequenced on a NovaSeq 6000 (Illumina) S2 × 150 bp flow cell.

### Data processing

#### Reads mapping

In the first analysis part, reads were mapped using BWA mem software (v0.7.15, Li, 2013) on the Human reference genome (hg19 assembly) using default parameters. As a second quality control, statistics regarding the mapping (percentage of aligned reads total and falling into the capture, percentage of PCR duplicates) and the capture coverage were produced using a combination of SAMtools, flagstat, BEDtools coverage and PicardTools MarkDuplicates.

#### Variant calling

Variant calling of both single nucleotide variations (SNVs) and small insertion/deletions (indels) was then performed on the processed alignment files using a combination of the SAMtools mpileup^52^ and *VarScan2* *mpileup2cns* (v2.4.3)^53^.

#### Annotations

Annotations from several databases [RefSeq, dbsnp v150, COSMIC v86, 1000g project 08/2015 version, ESP6500, gnomAD (all and ethnies), ICGC v21, and dbnsfp v35 predictions] were provided by ANNOVAR to annotate small variants^54^. Only the RefSeq database was used for intermediate-indel. During this step, all variants present in − 10/ + 10 bp of each exon junction were defined as splicing.

#### Coverage quality control

A more detailed notion of each gene percentage, per barcode, covered by at least 100×, in the processed alignments, was also provided using a combination of awk, SAMtools mpileup, BEDtools intersect, multiinter and merge. Bases covered by less than 100× were reported per barcode using the same strategy. Genes belonging to patient pathology were tagged in these two files to facilitate the search for genes of interest that might be badly covered.

### Bioinformatics analyses

#### Variant selection algorithm

Stringent selection algorithm was applied to remove a maximum of irrelevant variants. We considered a minimal allelic ratio of 5% and a maximal frequency in the population of 0.1%. Only truncating mutations (frameshift deletion and insertion, stopgain, splicing alteration and hotspot mutations from Cancer Hotspot database) with a minimal coverage of 200 reads were retained for tumor suppressor gene variants. All missense variants known to be hotspot mutations from Cancer Hotspot database and no minimal coverage were retained for oncogenes variants^55^. For genes classified as both oncogenes and TSG (such as *NOTCH1*) or with known missense hotspots (like *TP53*), truncating mutations with a minimal coverage of 200, and known hotspots mutations with no minimal coverage were selected.

#### Oncoprints

Oncoprints were drawn using the ComplexHeatmap package and were performed with the Maftools package for 4.00 version of R.

#### Tumor mutational burden

TMB was defined as the number of non-synonymous somatic mutations (SNVs and small indels) per mega-base in coding regions (mut/Mb). Coding variants (except for intronic splicing ones, therefore exons-only which represent 1.59 Mb), without synonymous nor polymorphisms (> 0.1% minor allele frequency [MAF]) and recurrent variants covered enough (not tagged as Low_Depth) were considered in all those calculations. Because the median and range of mutational load have been shown to vary across tumor types, we focused on the top 10% patients with the highest TMB scores and determined the log-rank p value for difference in survival among the 10% top TMB and 90% bottom TMB subgroups of patients.

### RNA sequencing

#### 3′ Tag-Seq protocol

High-throughput 3′ Tag RNAseq was performed on frozen and formalin fixed and paraffin embedded (FFPE) tumor RNA (100 ng). We used the QuantSeq 3' mRNA-Seq Library Prep Kit (for Illumina) from Lexogen company. In contrast to traditional RNA-seq, which generates sequencing libraries for the whole transcript, 3′ Tag-Seq only generates a single initial library molecule per transcript, complementary to 3′ end sequences.

### Data processing

RNAseq data was analyzed using the bioinformatics pipeline developed in Institut Curie and available at https://github.com/bioinfo-pf-curie/RNAseq. Briefly, raw sequencing reads were checked for quality, trimmed for potential adapter sequences and aligned on the Human reference genome with the STAR software. State-of-the-art quality controls such as library complexity, alignment statistics, duplication level, reads annotation were performed in order to ensure a high-quality of RNAseq samples. Gene expression quantification was performed with the featureCounts tool on the coding genes annotation extracted from the Gencode project.

### Bioinformatics analyses

Unsupervised clustering k-sparse methods^56^ with number of cluster (k) equal to two of bulk-tissue RNAseq data was used. A batch effect-correction algorithm (https://github.com/Jfortin1/ComBatHarmonization) was then applied in an attempt to mitigate the dataset technical heterogeneity. The ComBat method, initially described in genomics, is a realignment method used to deal with the batch effect. In genomics, the batch effect represents individual variations due only to variations between technicians, laboratories, or the days of manipulations.

#### Identification of differentially expressed genes

We computed ratios of surgery/biopsy normalized gene expression levels as measured by RNAseq, with “biopsy” referring to treatment-naïve tumor samples, and “surgery” the paired post-afatinib ones. The Mann–Whitney *U* test was performed to identify variability of expression ratios between two response (responders versus non-responders) groups. A cut-off of p-value < 0.05 was applied to select the most differentially expressed genes in the context of therapy.

#### Pathway enrichment analyses

Over Representation Analysis (ORA) was used for enrichment analysis^57^. Pathway enrichment was computed using the Kyoto Encyclopedia of Genes (KEGG) database (https://www.kegg.jp/kegg/kegg1.html) with hypergeometric test false discovery rate ≤ 0.1. Network-based pathway enrichment analyses were performed using ratios of surgery/biopsy gene expression levels across responders and non-responders in the bulk-tissue RNAseq data. In the bulk-tissue, the differentially expressed genes in the context of therapy that had a p-value < 0.05 were selected as input for pathway analyses. ORA was performed separately by the direction of change in gene expression in order to identify gene sets that are over-represented when we only consider genes that are up- or down-regulated in one condition relative to another. KEGG datasets were retrieved from the R package software clusterProfiler v4.5.0^58^.

#### Deconvolution of tumor microenvironment immune cells

The R package software quanTIseq^59^ was applied to bulk RNAseq data from paired pre- and post-tumor samples to quantify the fractions of ten immune cell types, including B cells, M1 and M2 macrophages, monocytes, neutrophils, natural killer cells, non-regulatory CD4+ T cells, CD8+ T cells, T_reg_ cells and myeloid dendritic cells. quanTIseq also estimated the proportion of uncharacterized cells, namely cells that were present in the cell mixture of interest but were not represented in the signature matrix (e.g., cancer cells). Importantly, quanTIseq scores are proportional to the amount of each cell population in the total sample, thus allowing intra- and inter-sample comparison for each population.

### High throughput protein analysis

High throughput protein analysis used RPPA methods (Supplementary Fig. 10). The samples were processed as previously described^60^ and printed onto nitrocellulose covered slides (Supernova, Grace Biolabs) using a dedicated arrayer (2,470 arrayer, Aushon Biosystems). Five serial dilutions, starting at 2000 mg/ml and two technical replicates per dilution were printed for each sample. Arrays were labelled with 77 specific, or without primary antibody (as negative control), as previously described^60^. All primary antibodies used in RPPA have been previously tested by Western Blotting to assess their specificity for the protein of interest. Raw data were normalized using Normacurve^61^, which normalizes for fluorescent background per spot, a total protein stain and potential spatial bias on the slide. Next, each RPPA slide was median centered and scaled (divided by median absolute deviation). We then corrected for remaining sample loading effects individually for each array by correcting the dependency of the data for individual arrays on the median value of each sample, over all 77 arrays, using a linear regression.

The panel covered post translationally modified cancer pathway proteins, including phosphorylated and non-phosphorylated proteins derived from cell cycle, cell migration, genome integrity, immunity, metabolism, JAK/STAT signaling, MAPK signaling, NF-kappa B signaling, PI3K/AKT/mTOR signaling, receptor tyrosine kinase signaling, and Wnt/β-catenin signaling pathways (Supplementary Table 8).

### Statistical analyses

The sample size was calculated to have a 90% power to detect a difference in treatment effect between biomarker positive versus biomarker negative patients. The treatment effect was defined as the difference in the tumor size evolution between the two arms. Since the observed mean tumor reduction under afatinib was less than the anticipated threshold of 20%^62^, correlative biomarkers analyses were done on the basis of the metabolic response and thus remained mainly exploratory.

All statistical analyses were performed using R software. Comparisons between groups were assessed using the Wilcoxon rank-sum test for quantitative variables, and the Fisher test for qualitative variables. For post-hoc analyses, univariate and multivariate Cox proportional hazard models were performed to identify prognostic biomarkers. The results are presented as hazard ratios (HR) and 95% confidence intervals (CIs). Survival curves were obtained with Kaplan–Meier estimates and compared using the log-rank test. The level of significance was set at *P* < 0.05. Correction for multiple testing was not applied.

### Supplementary Information

Supplementary Figure 1.

Supplementary Figure 2.

Supplementary Figure 3.

Supplementary Figure 3.

Supplementary Figure 4.

Supplementary Figure 4.

Supplementary Figure 5.

Supplementary Figure 5.

Supplementary Figure 6.

Supplementary Figure 7.

Supplementary Figure 8.

Supplementary Figure 8.

Supplementary Figure 9.

Supplementary Figure 10.

Supplementary Legends.

Supplementary Information 1.

Supplementary Information 2.

Supplementary Information 3.

Supplementary Table 1.

Supplementary Table 2.

Supplementary Table 3.

Supplementary Table 4.

Supplementary Table 5.

Supplementary Table 6.

Supplementary Table 7.

Supplementary Table 8.

## Data Availability

Original files and raw data files from the GEP11 PREDICTOR clinical trial will be made available from the corresponding authors upon reasonable request. The data are not publicly available due to information that could compromise the privacy of the research participants. All processed data used for the analyses are available in the Supplementary Materials.

## Abbreviations

AE: Adverse event
CI: Confidence interval
CTCAE: National Cancer Institute Common Terminology Criteria for Adverse Events
DNAseq: DNA sequencing
DRAGON: Detection of relevant alterations in genes involved in oncogenetics
FFPE: Frozen and formalin fixed and paraffin embedded
HER: Human epidermal growth factor receptor
HR: Hazard ratio
HNSCC: Head and neck squamous cell carcinoma
EGFR: Epidermal growth factor receptor
FDG-PET: FDG-positron emission tomography
FDR: False discovery rate
GSEA: Gene set enrichment analysis
IQR: Interquartile range
mAb: Monoclonal antibody
NSCL: Non-small cell lung cancer
NGS: Next generation sequencing
ORA: Over representation analysis
OS: Overall survival
PERCIST: Positron emission tomography response criteria in solid tumours
PFS: Progression-free survival
RECIST1.1: Response evaluation criteria in solid tumors version 1.1
RNAseq: RNA sequencing
RPPA: Reverse phase protein arrays
TKI: Tyrosine kinase inhibitor
TME: Tumor micro environment
TSG: Tumor suppressor gene
TMB: Tumor mutational burden
