# Supplementary figures and images for: Randomized phase II study of preoperative afatinib in untreated head and neck cancers: predictive and pharmacodynamic biomarkers of activity
Source: Sci Rep. 2023 Dec 18;13:22524. doi: 10.1038/s41598-023-49887-4 (PMC10728082; doi:10.1038/s41598-023-49887-4)

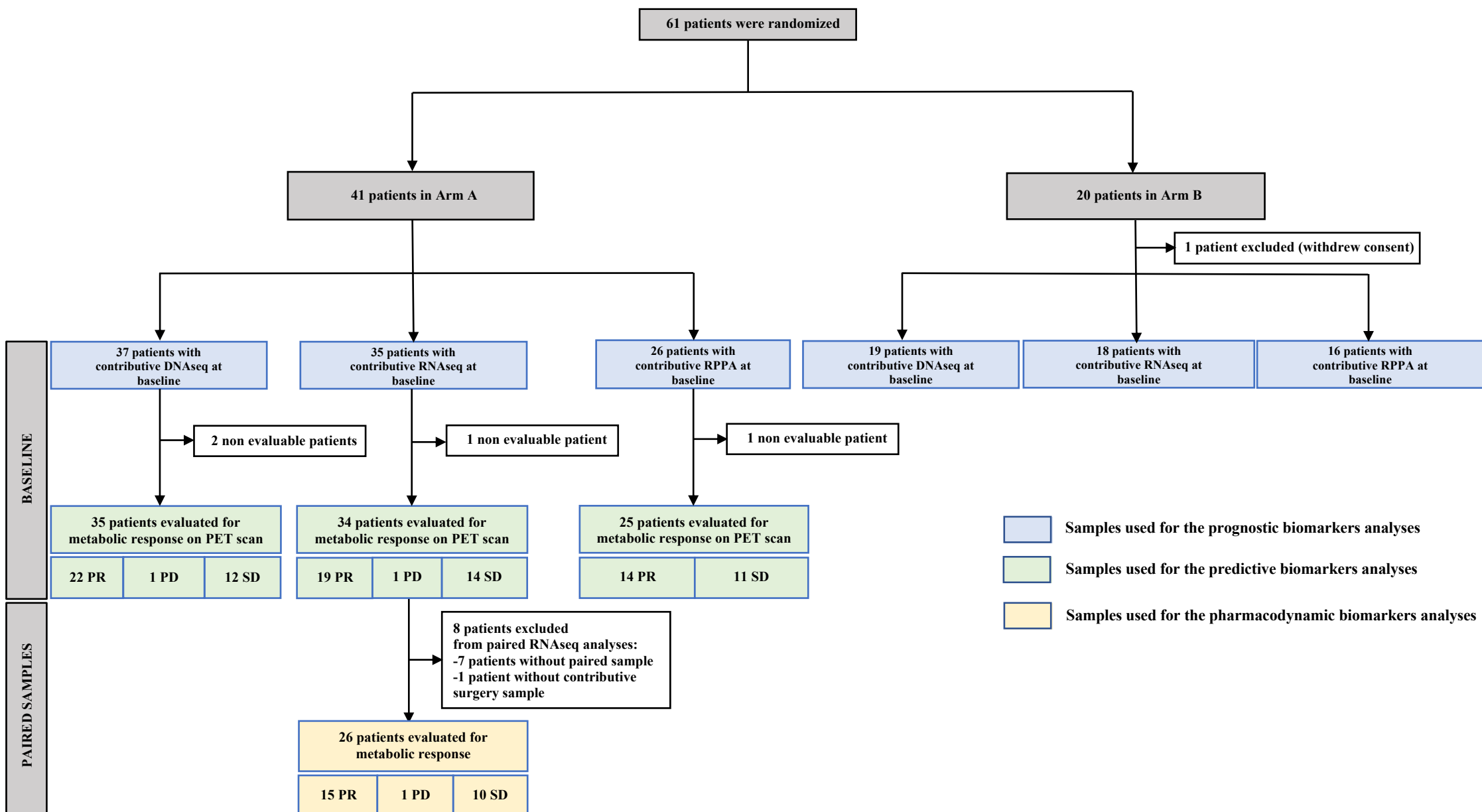

Supplementary Figure 1

Supplement: Supplementary file 1 — Supplementary Figure 1. [file 41598_2023_49887_MOESM1_ESM.pdf]

All patients (N=56)

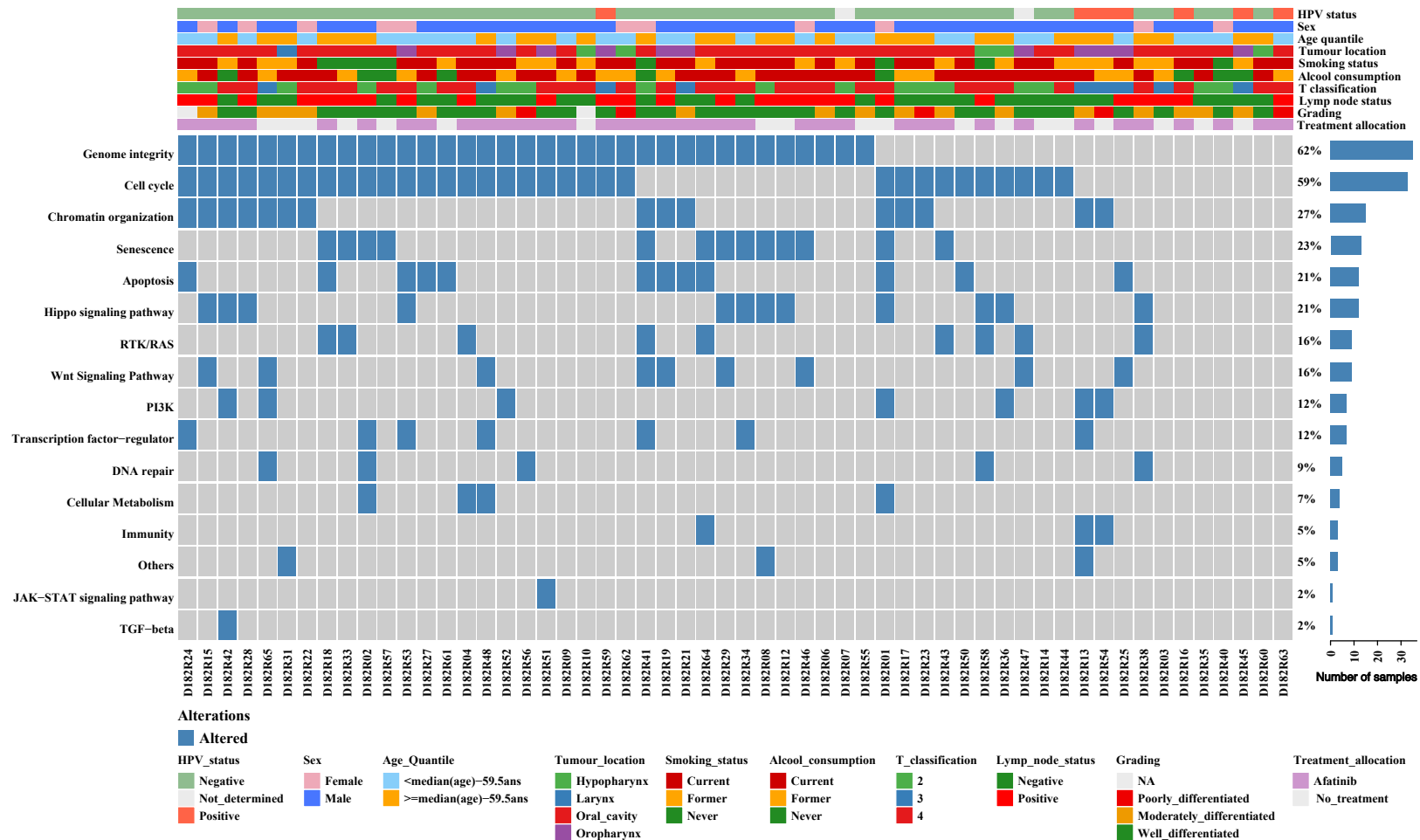

Supplementary Figure 2

Supplement: Supplementary file 2 — Supplementary Figure 2. [file 41598_2023_49887_MOESM2_ESM.pdf]

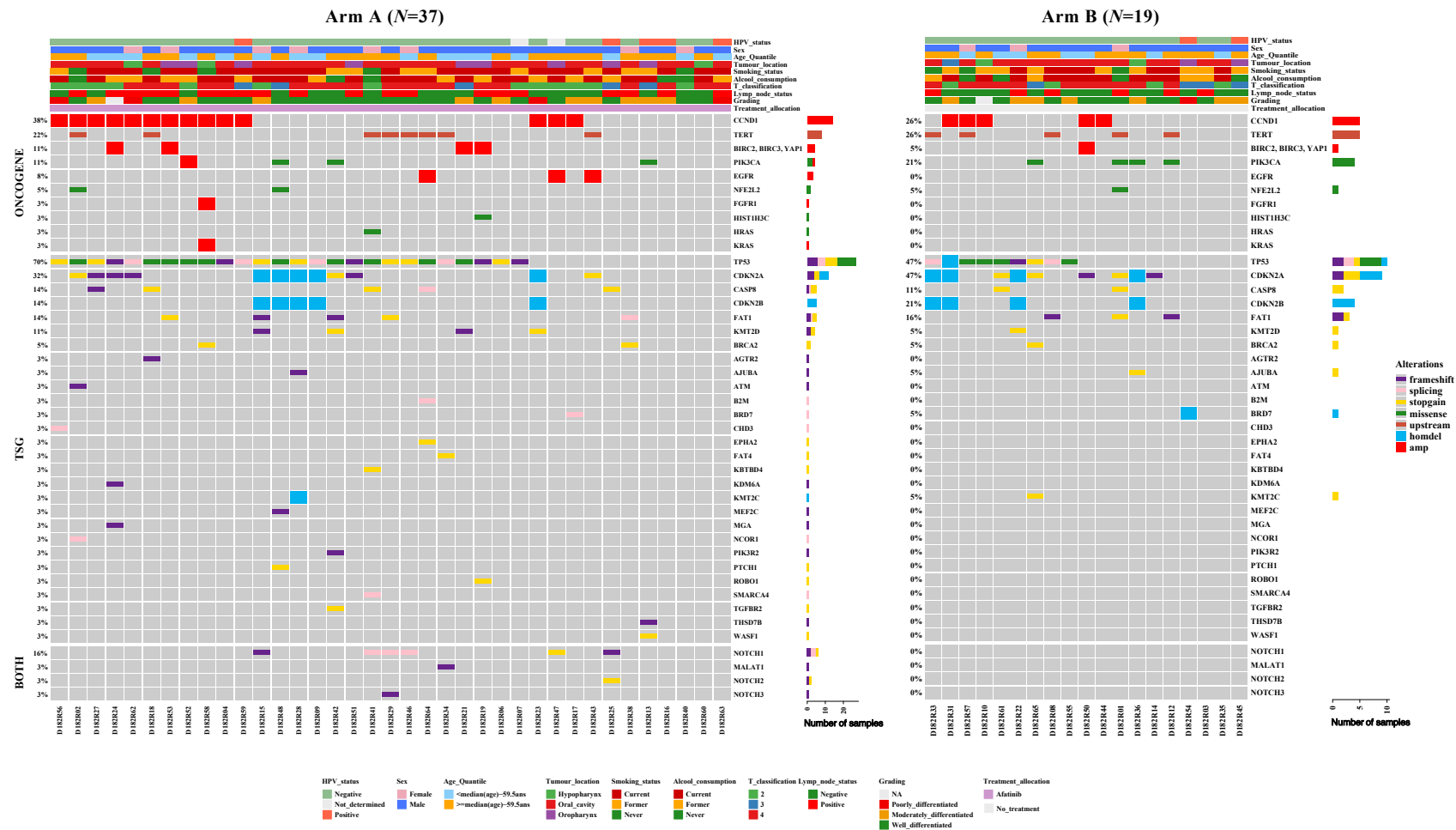

Supplementary Figure 3A

Supplement: Supplementary file 3 — Supplementary Figure 3. [file 41598_2023_49887_MOESM3_ESM.pdf]

Supplementary Figure 3B

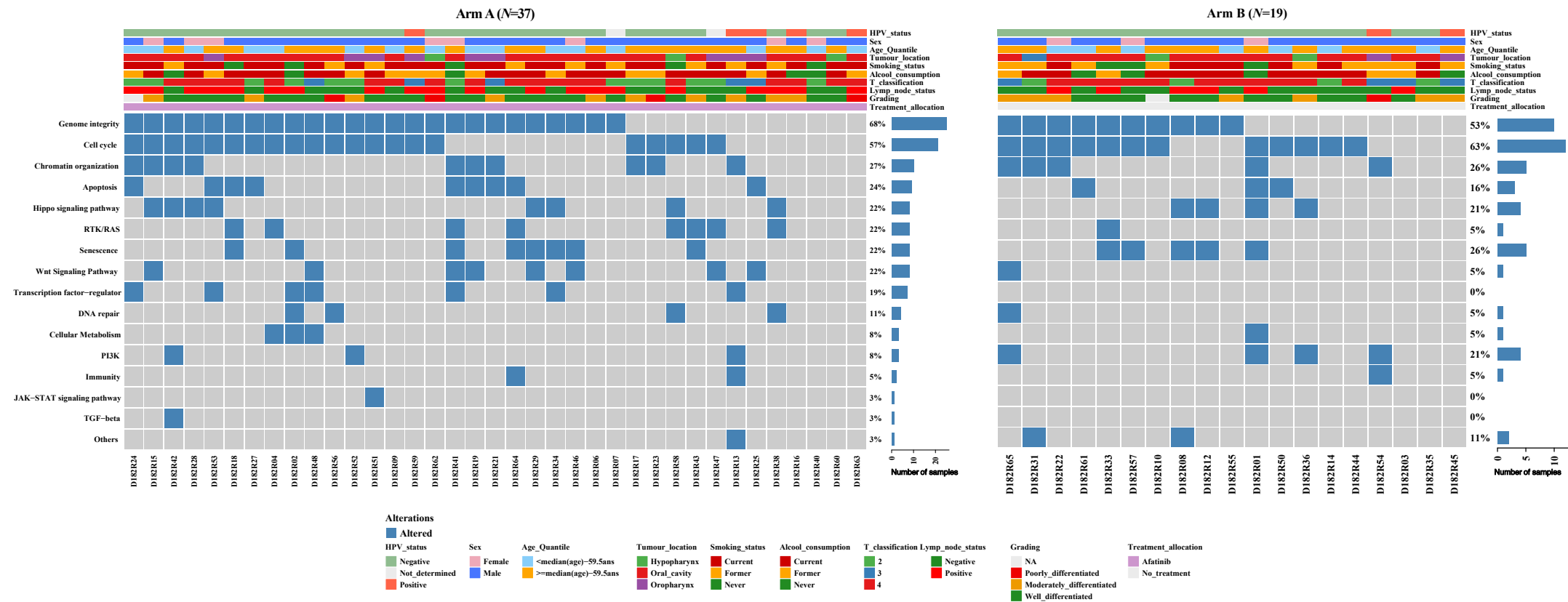

Supplement: Supplementary file 4 — Supplementary Figure 3. [file 41598_2023_49887_MOESM4_ESM.pdf]

NF-KAPPA B SIGNALING PATHWAY

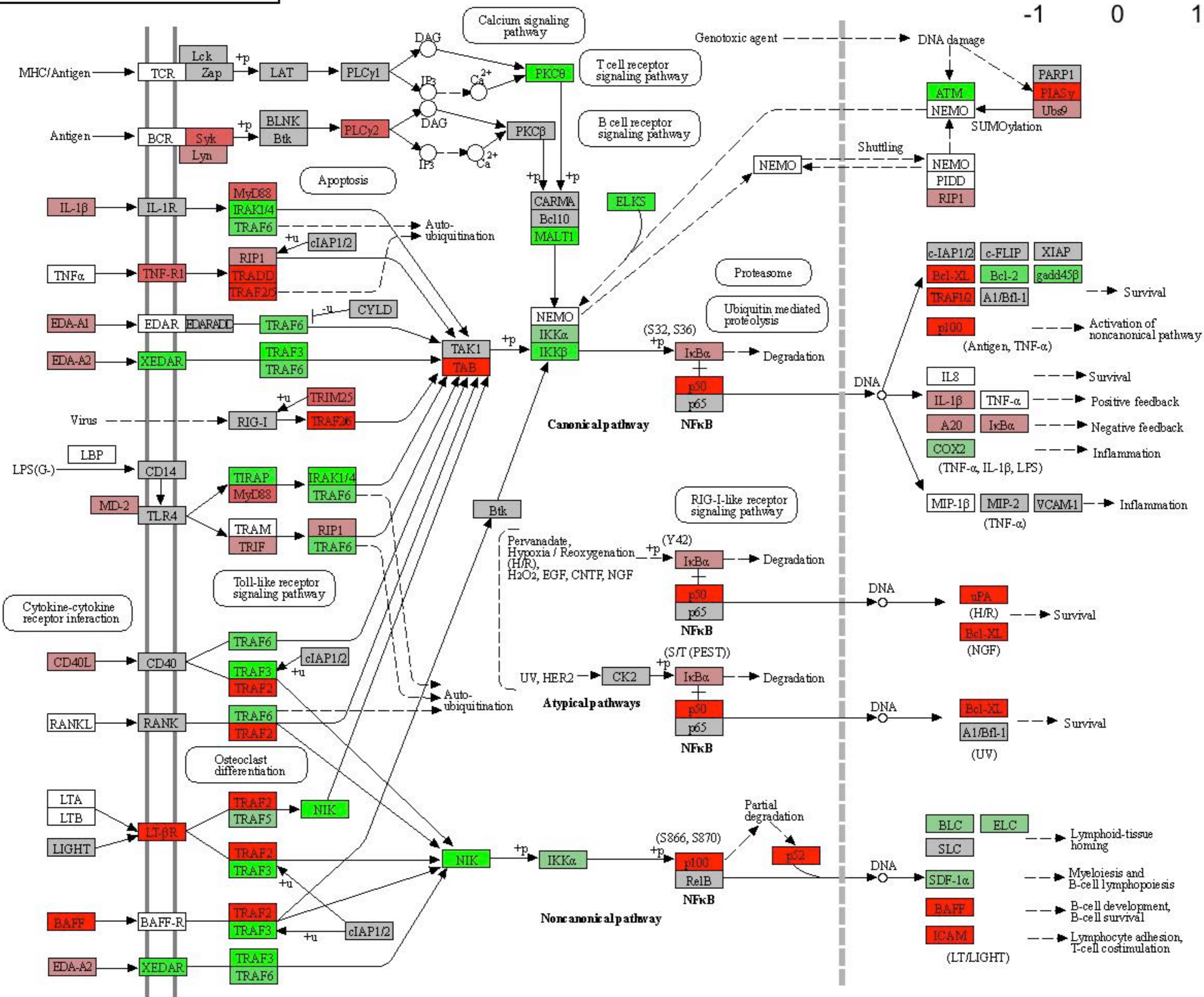

Supplement: Supplementary file 10 — Supplementary Figure 7. [file 41598_2023_49887_MOESM10_ESM.pdf]

Supplementary Figure 8A

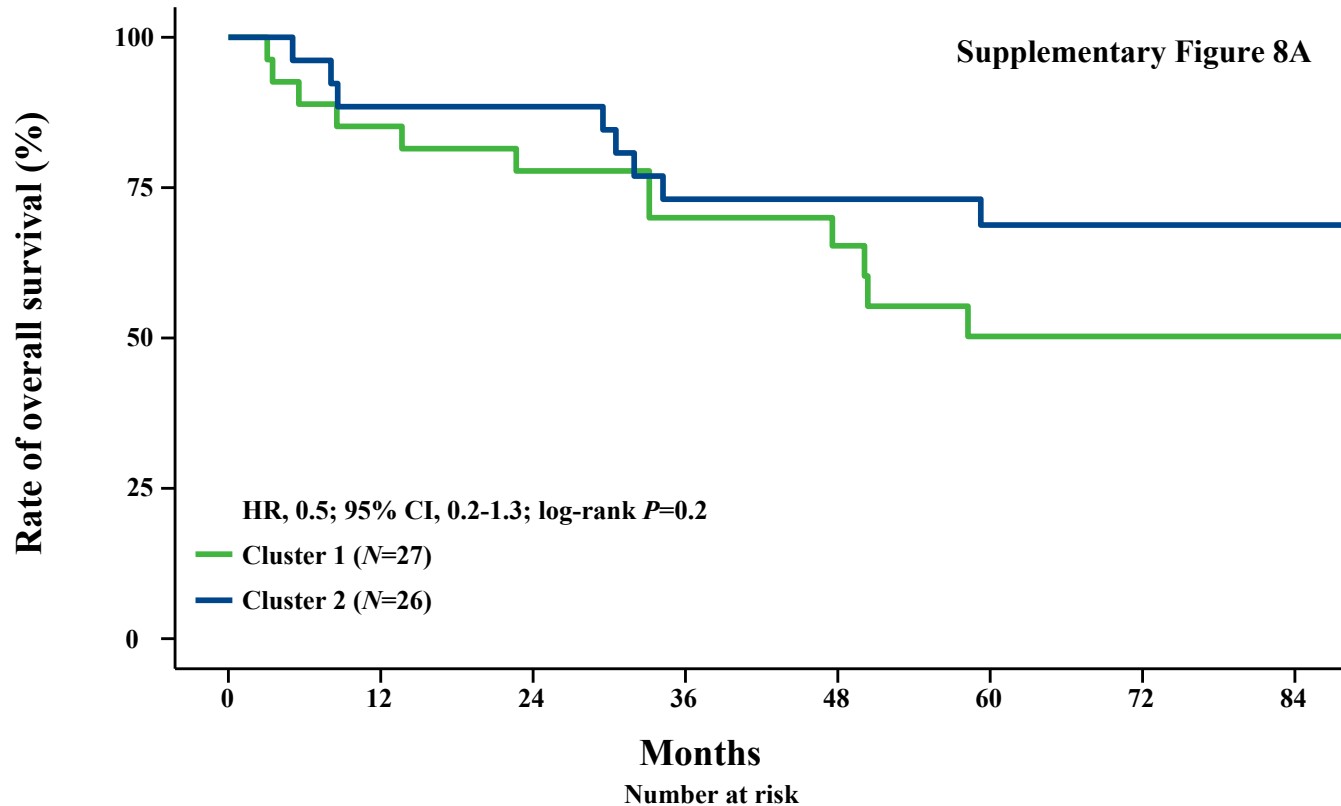

|                                |    |    |    |    |    |    |    |    |
|--------------------------------|----|----|----|----|----|----|----|----|
| Cluster 1                      | 27 | 23 | 20 | 18 | 14 | 9  | 8  | 7  |
| Cluster 2                      | 26 | 23 | 23 | 19 | 18 | 16 | 14 | 8  |
| Cumulative number of censoring |    |    |    |    |    |    |    |    |
| Cluster 1                      | 0  | 0  | 1  | 1  | 4  | 6  | 7  | 8  |
| Cluster 2                      | 0  | 0  | 0  | 0  | 1  | 2  | 4  | 10 |

Supplement: Supplementary file 11 — Supplementary Figure 8. [file 41598_2023_49887_MOESM11_ESM.pdf]

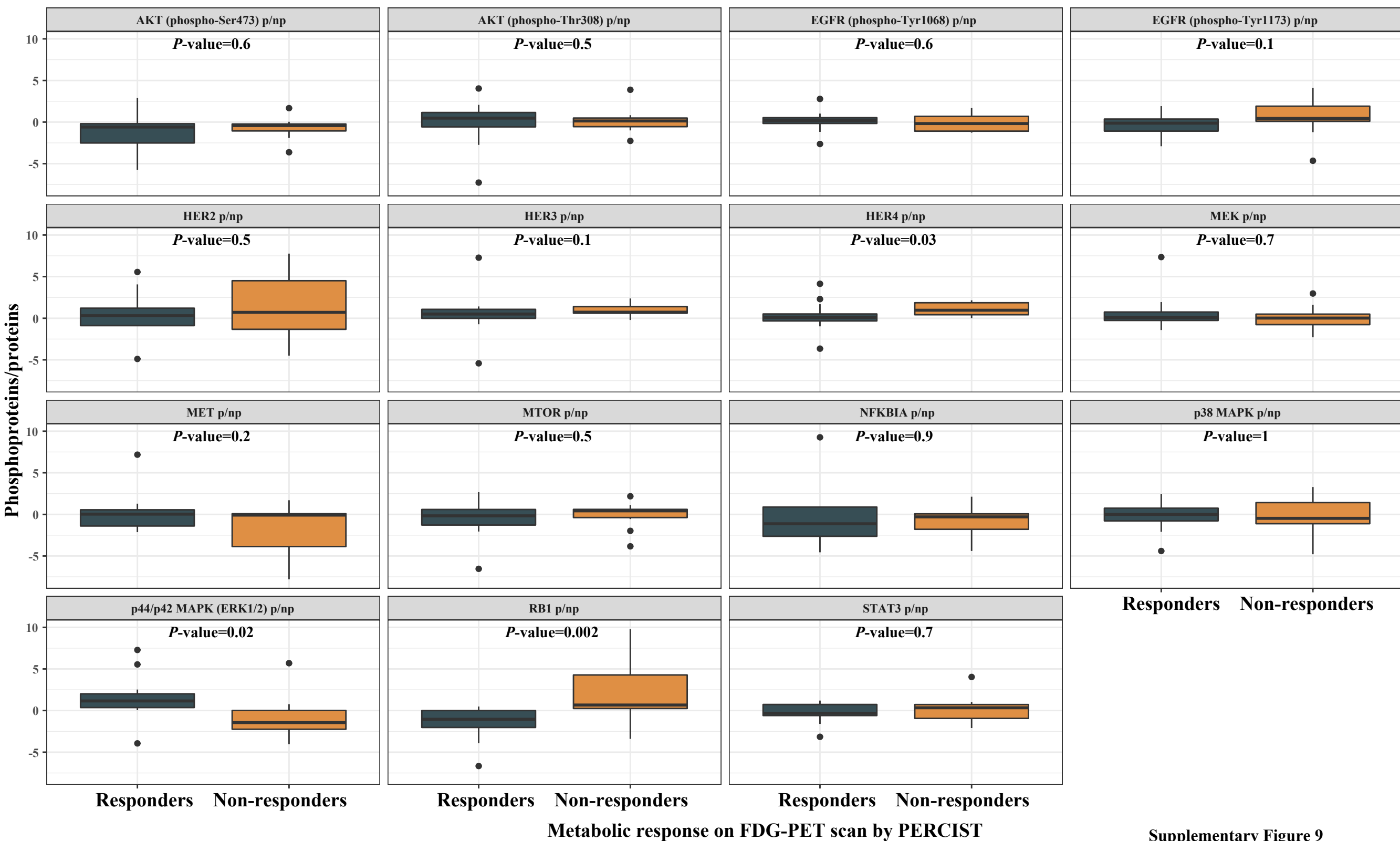

Supplementary Figure 9

Supplement: Supplementary file 13 — Supplementary Figure 9. [file 41598_2023_49887_MOESM13_ESM.pdf]

**A**

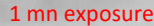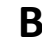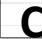

## Supplementary Figure 10

Supplement: Supplementary file 14 — Supplementary Figure 10. [file 41598_2023_49887_MOESM14_ESM.pdf]
