# Supplementary material for: Randomized phase II study of preoperative afatinib in untreated head and neck cancers: predictive and pharmacodynamic biomarkers of activity
Source: Sci Rep. 2023 Dec 18;13:22524. doi: 10.1038/s41598-023-49887-4 (PMC10728082; doi:10.1038/s41598-023-49887-4)
Supplement: Supplementary file 5 — Supplementary Figure 4. [file 41598_2023_49887_MOESM5_ESM.pdf]

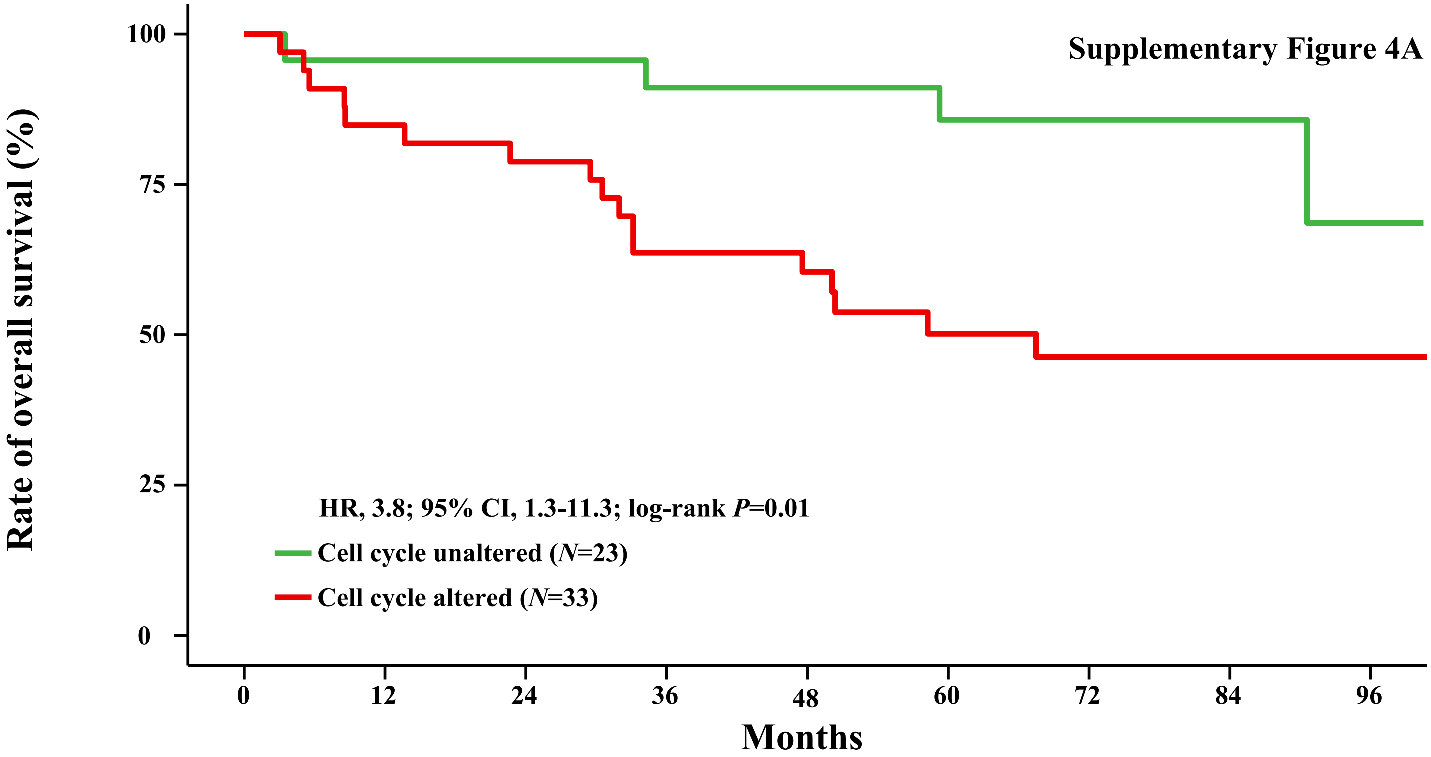

Number at risk

	0	12	24	36	48	60	72	84	96
Cell cycle unaltered	23	22	21	20	17	16	15	11	2
Cell cycle altered	33	28	26	21	19	13	11	6	1

Cumulative number of censoring

	0	12	24	36	48	60	72	84	96
Cell cycle unaltered	0	0	1	1	4	4	5	9	17
Cell cycle altered	0	0	0	0	1	4	5	10	15
