# Supplementary material for: Randomized phase II study of preoperative afatinib in untreated head and neck cancers: predictive and pharmacodynamic biomarkers of activity
Source: Sci Rep. 2023 Dec 18;13:22524. doi: 10.1038/s41598-023-49887-4 (PMC10728082; doi:10.1038/s41598-023-49887-4)
Supplement: Supplementary file 6 — Supplementary Figure 4. [file 41598_2023_49887_MOESM6_ESM.pdf]

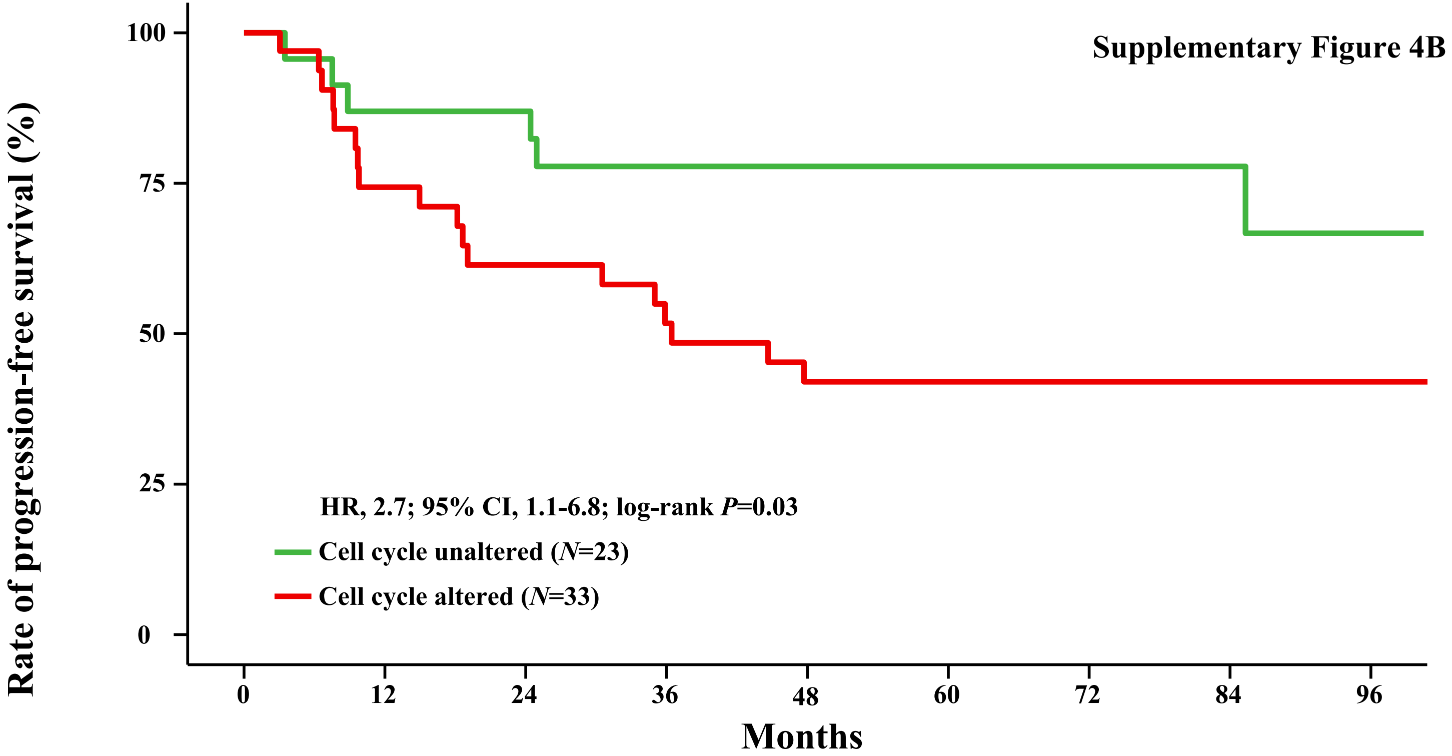

Number at risk

	0	12	24	36	48	60	72	84	96
Cell cycle unaltered	23	20	19	16	13	13	12	9	2
Cell cycle altered	33	23	19	16	13	9	7	3	1

Cumulative number of censoring

	0	12	24	36	48	60	72	84	96
Cell cycle unaltered	0	0	1	2	5	5	6	9	15
Cell cycle altered	0	2	2	2	2	6	8	12	14
