# Supplementary material for: Randomized phase II study of preoperative afatinib in untreated head and neck cancers: predictive and pharmacodynamic biomarkers of activity
Source: Sci Rep. 2023 Dec 18;13:22524. doi: 10.1038/s41598-023-49887-4 (PMC10728082; doi:10.1038/s41598-023-49887-4)
Supplement: Supplementary file 7 — Supplementary Figure 5. [file 41598_2023_49887_MOESM7_ESM.pdf]

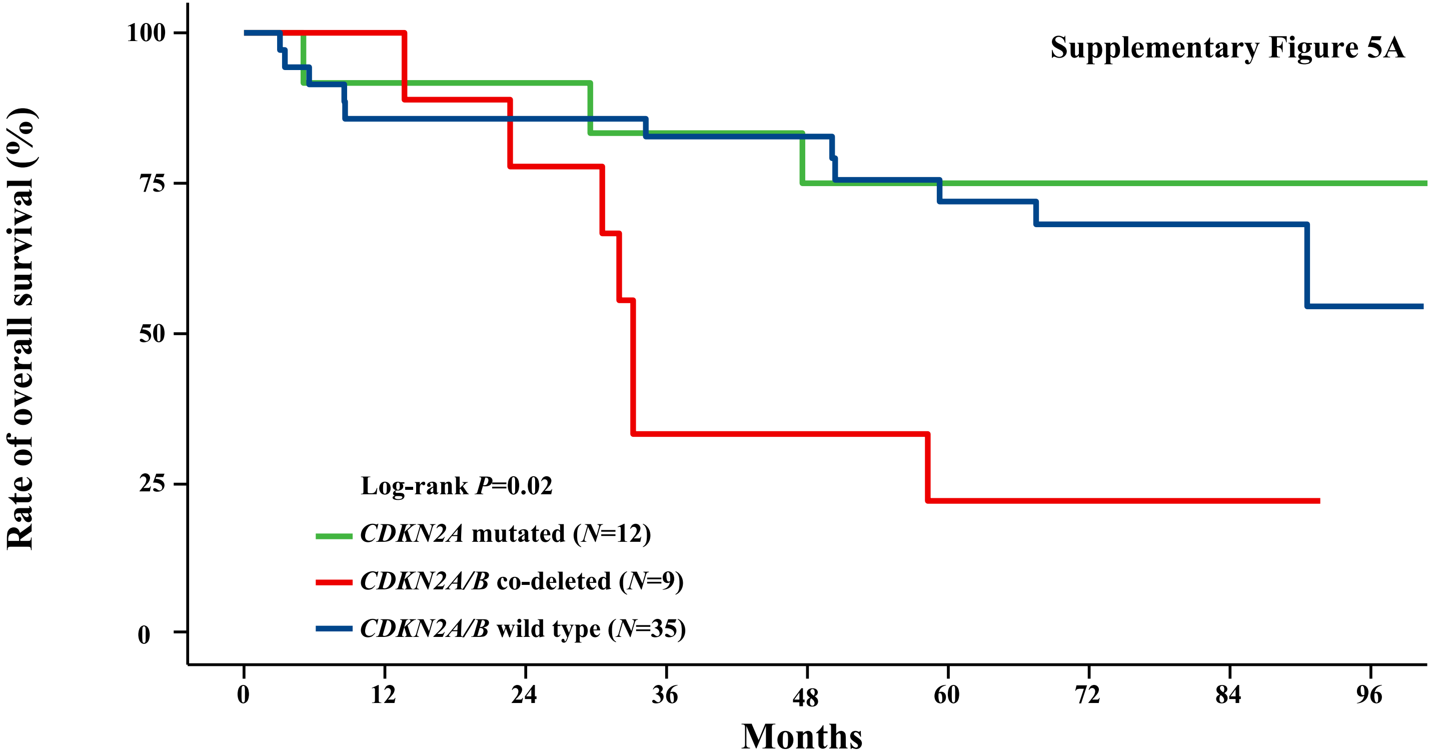

Number at risk

	0	12	24	36	48	60	72	84	96
CDKN2A mutated	12	11	11	10	9	7	6	4	1
CDKN2A/B co-deleted	9	9	7	3	3	2	2	2	0
CDKN2A/B wild type	35	30	29	28	24	20	18	11	2

Cumulative number of censoring

	0	12	24	36	48	60	72	84	96
CDKN2A mutated	0	0	0	0	0	2	3	5	8
CDKN2A/B co-deleted	0	0	0	0	0	0	0	0	2
CDKN2A/B wild type	0	0	1	1	5	6	7	14	22
