# Supplementary material for: Randomized phase II study of preoperative afatinib in untreated head and neck cancers: predictive and pharmacodynamic biomarkers of activity
Source: Sci Rep. 2023 Dec 18;13:22524. doi: 10.1038/s41598-023-49887-4 (PMC10728082; doi:10.1038/s41598-023-49887-4)
Supplement: Supplementary file 8 — Supplementary Figure 5. [file 41598_2023_49887_MOESM8_ESM.pdf]

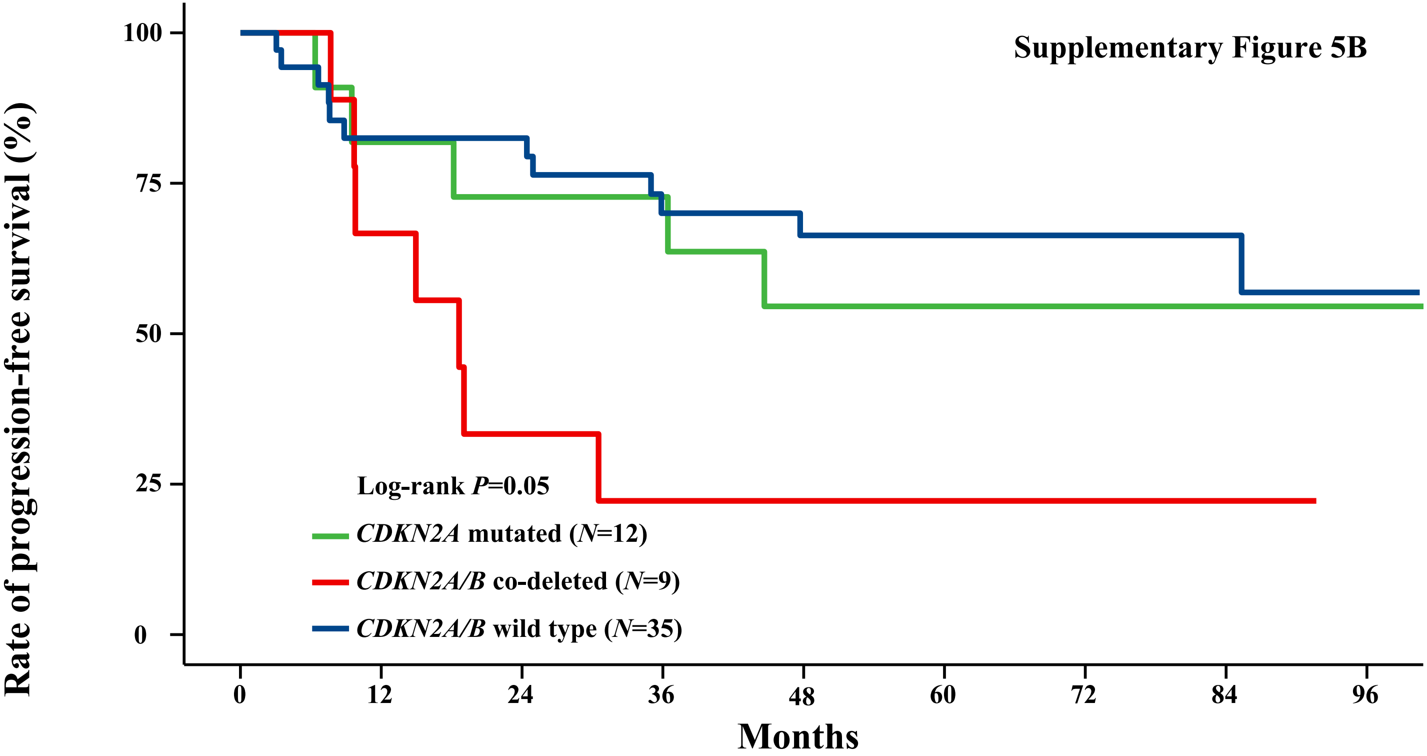

Number at risk

CDKN2A mutated	12	9	8	8	6	4	3	2	1
CDKN2A/B co-deleted	9	6	3	2	2	1	1	1	0
CDKN2A/B wild type	35	28	27	22	18	17	15	9	2

Cumulative number of censoring

CDKN2A mutated	0	1	1	1	1	3	4	5	6
CDKN2A/B co-deleted	0	0	0	0	0	1	1	1	2
CDKN2A/B wild type	0	1	2	3	6	7	9	15	21
