# Supplementary material for: Randomized phase II study of preoperative afatinib in untreated head and neck cancers: predictive and pharmacodynamic biomarkers of activity
Source: Sci Rep. 2023 Dec 18;13:22524. doi: 10.1038/s41598-023-49887-4 (PMC10728082; doi:10.1038/s41598-023-49887-4)
Supplement: Supplementary file 9 — Supplementary Figure 6. [file 41598_2023_49887_MOESM9_ESM.pdf]

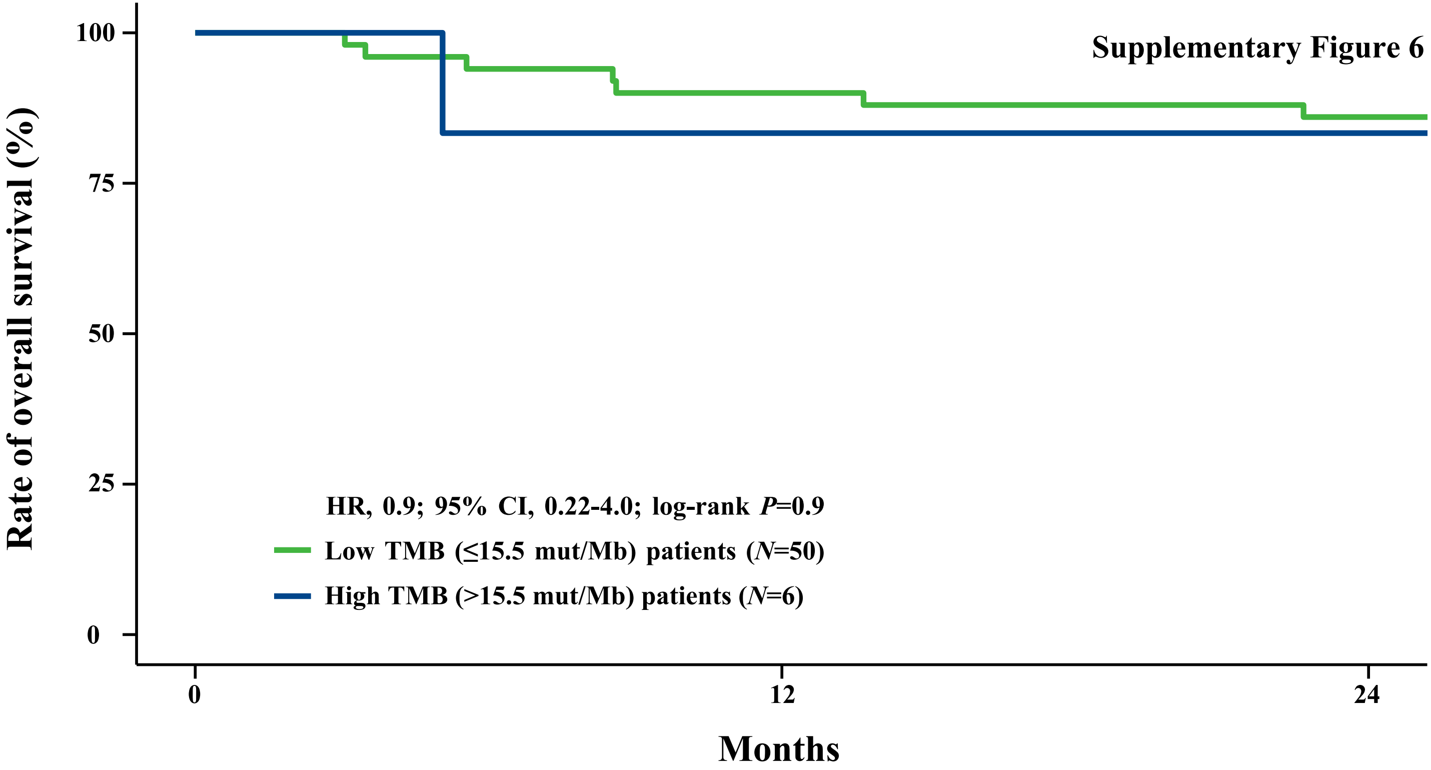


**Number at risk**

| | 0 | 12 | 24 |
|---|---|---|---|
| Low TMB patients | 50 | 45 | 42 |
| High TMB patients | 6 | 5 | 5 |

**Cumulative number of censoring**

| | 0 | 12 | 24 |
|---|---|---|---|
| Low TMB patients | 0 | 0 | 1 |
| High TMB patients | 0 | 0 | 0 |
