# Supplementary material for: Randomized phase II study of preoperative afatinib in untreated head and neck cancers: predictive and pharmacodynamic biomarkers of activity
Source: Sci Rep. 2023 Dec 18;13:22524. doi: 10.1038/s41598-023-49887-4 (PMC10728082; doi:10.1038/s41598-023-49887-4)
Supplement: Supplementary file 12 — Supplementary Figure 8. [file 41598_2023_49887_MOESM12_ESM.pdf]

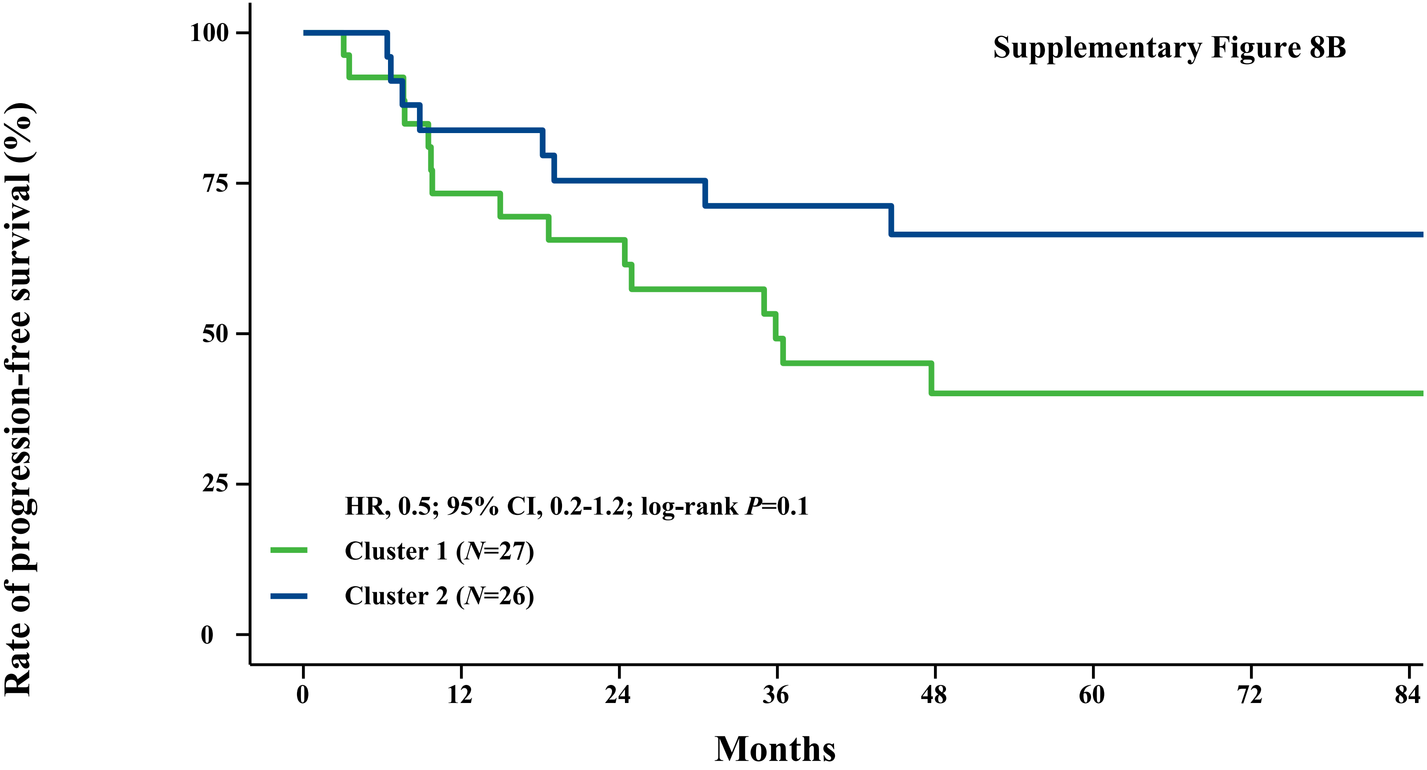

Number at risk

Cluster 1	27	19	16	12	8	5	4	3
Cluster 2	26	20	18	16	14	13	11	7

Cumulative number of censoring

Cluster 1	0	1	2	2	4	7	8	9
Cluster 2	0	2	2	3	4	5	7	11
