# Supplementary material for: Randomized phase II study of preoperative afatinib in untreated head and neck cancers: predictive and pharmacodynamic biomarkers of activity
Source: Sci Rep. 2023 Dec 18;13:22524. doi: 10.1038/s41598-023-49887-4 (PMC10728082; doi:10.1038/s41598-023-49887-4)
Supplement: Supplementary file 15 — Supplementary Legends. [file 41598_2023_49887_MOESM15_ESM.docx]

**Supplementary Figure legends**

**Supplementary Figure 1** Workflow of translational analyses

PR, Partial Response; PD, Progressive Disease; SD, Stable Disease

**Supplementary Figure 2** Overview of altered signaling pathways in the whole patient population

**Supplementary Figure 3** Overview of gene alterations (A), and altered signaling pathways (B) in Arm A (*N*=37) and Arm B (*N*=19)

**Supplementary Figure 4** Prognostic value of cell cycle alterations on overall survival (A), and progression-free survival (B)

**Supplementary Figure 5** Prognostic value of *CDKN2A/B* alterations on overall survival (A), and progression-free survival (B)

**Supplementary Figure 6** Prognostic value of tumor mutational burden (TMB) on overall survival in patients with high (*N*=6) and low (*N*=50) TMB

**Supplementary Figure 7** Mapping of differentially expressed genes in the KEGG NF-kappa B signaling between non-responders and responders. The color scale is representative of log2 fold change in non-responders *versus* responders.

KEGG, Kyoto Encyclopedia of Genes datasets

**Supplementary Figure 8** Prognostic value of the two clusters of patients (Cluster 1, *N*=27; Cluster 2, *N*=26) identified by unsupervised analysis on bulk gene expression data on overall survival (A), and progression-free survival (B)

**Supplementary Figure 9** Relationship between phosphorylation levels of human epidermal growth factor receptors and downstream proteins and metabolic response on FDG-PET scan according to PERCIST (responders, *N*=14; non-responders, *N*=11)

p/np, phosphoproteins/proteins

**Supplementary Figure 10** Workflow of Reverse Phase Protein Array (RPPA) analyses.

A: Raw data quality control. Top: Antibody spot intensities were plotted against serial dilutions for each antibody (one curve is representative of one sample). Middle: Antibody spot intensities were compared to negative control slides (blue-colored boxplot corresponds to one antibody tested in all samples, while black-colored boxplot corresponds to the negative control). Bottom: All primary antibody used in RPPA assay were tested by western blotting to assess their specificity for the protein of interest. B: Data normalization. Top: Antibody spot intensities were normalized for fluorescent background per spot, total protein stain and potential spatial bias on the slide. Middle and bottom: Each RPPA slide was median corrected, making samples more comparable. C: Exploratory RPPA analyses. Exploratory analyses included unsupervised clustering on protein expression data (top and middle) and comparative analysis by metabolic response to afatinib (bottom).
