# Supplementary material for: Randomized phase II study of preoperative afatinib in untreated head and neck cancers: predictive and pharmacodynamic biomarkers of activity
Source: Sci Rep. 2023 Dec 18;13:22524. doi: 10.1038/s41598-023-49887-4 (PMC10728082; doi:10.1038/s41598-023-49887-4)
Supplement: Supplementary file 19 — Supplementary Table 1. [file 41598_2023_49887_MOESM19_ESM.docx]

**Supplementary Table 1.** Summary of adverse events by treatment allocation

| Adverse drug reactions  n (%) | Grade^a^ | Arm A  (*N*=41) | Arm B  (*N*=18) |
| --- | --- | --- | --- |
| Gastrointestinal disorders |  |  |  |
| Diarrhea^¶^ | 1 | 1 (2.4%) | 0 |
|  | 2 | 4 (9.8%) | 0 |
|  | 4 | 1 (2.4%) | 0 |
| Oral mucositis^¶^ | 1 | 2 (4.9%) | 0 |
|  | 2 | 1 (2.4%) | 0 |
|  | 3 | 2 (4.9%) | 0 |
| Dysphagia^¶^ | 1 | 1 (2.4%) | 0 |
|  | 2 | 1 (2.4%) | 0 |
| Nausea^¶^ | 2 | 1 (2.4%) | 0 |
| Dry mouth | 1 | 1 (2.4%) | 0 |
| General disorders |  |  |  |
| Fatigue^¶^ | 1 | 1 (2.4%) | 0 |
|  | 2 | 1 (2.4%) | 0 |
|  | 3 | 1 (2.4%) | 0 |
| Fever | 3 | 1 (2.4%) |  |
| Infections and infestations |  |  |  |
| Folliculitis^¶^ | 1 | 1 (2.4%) | 0 |
|  | 4 | 1 (2.4%) | 0 |
| Sinusitis | 2 | 1 (2.4%) | 0 |
| Investigations |  |  |  |
| Alkaline phosphatase increase^¶^ | 2 | 1 (2.4%) | 0 |
| Blood and lymphatic system disorders |  |  |  |
| Anemia | 3 | 1 (2.4%) | 0 |
| Lymphocyte count decrease | 2 | 1 (2.4%) | 0 |
| Metabolism and nutrition disorders |  |  |  |
| Hyperglycemia^¶^ | 1 | 1 (2.4%) | 0 |
| Anorexia | 2 | 1 (2.4%) | 0 |
| Dehydration | 2 | 1 (2.4%) | 0 |
| Skin and subcutaneous tissue disorders |  |  |  |
| Acneiform rash^¶^ | 1 | 5 (12.2%) | 0 |
|  | 2 | 4 (9.8%) | 0 |
| Dry skin^¶^ | 1 | 4 (9.8%) | 0 |
|  | 2 | 2 (4.9%) | 0 |
| Multiforme erythema ^¶^ | 2 | 1 (2.4%) | 0 |
| Palmar-plantar syndrome^¶^ | 1 | 2 (4.9%) | 0 |
|  | 2 | 1 (2.4%) | 0 |
| Respiratory disorders |  |  |  |
| Laryngeal hemorrhage | 1 | 0 | 1 (5.6%) |
| Oropharyngeal pain | 1 | 1 (2.4%) | 0 |
|  | 2 | 0 | 1 (5.6%) |
| Neoplasms benign, malignant and unspecified |  |  |  |
| Tumor pain | 1 | 3 (7.3%) | 3 (16.7%) |
|  | 2 | 7 (17.1%) | 5 (27.8%) |
|  | 3 | 0 | 1 (5.6%) |
| Synchronous malignant neoplasm | 3 | 1 (2.4%) | 0 |
| Psychiatric disorders |  |  |  |
| Depression | 1 | 1 (2.4%) | 0 |
|  | 2 | 1 (2.4%) | 0 |
| Insomnia | 1 | 1 (2.4%) | 0 |
| Nervous system disorders |  |  |  |
| Stroke | 3 | 1 (2.4%) | 0 |
| Headache | 1 | 1 (2.4%) | 0 |
| Nervous sensory neuropathy | 1 | 1 (2.4%) | 0 |
|  | 2 | 1 (2.4%) | 0 |
| Dysarthria | 1 | 1 (2.4%) | 0 |
| Vascular disorders |  |  |  |
| Hypertension | 2 | 1 (2.4%) | 0 |

^a^Adverse events classified and graded using National Institute Common Terminology Criteria for Adverse Events version 4.1; ^¶^Afatinib-related adverse events
