# Supplementary material for: Randomized phase II study of preoperative afatinib in untreated head and neck cancers: predictive and pharmacodynamic biomarkers of activity
Source: Sci Rep. 2023 Dec 18;13:22524. doi: 10.1038/s41598-023-49887-4 (PMC10728082; doi:10.1038/s41598-023-49887-4)
Supplement: Supplementary file 20 — Supplementary Table 2. [file 41598_2023_49887_MOESM20_ESM.docx]

**Supplementary Table 2.** Predictive value of genomic alterations and altered molecular pathways

|  | Patients  n (%) | Efficacy on FDG-PET scan by PERCIST | | *P*-value |
| --- | --- | --- | --- | --- |
|  |  | **Non-responders**  **n (%)** | **Responders**  **n (%)** |  |
| Total | 35 (100%) | 13 (37%) | 22 (63%) |  |
| Genes |  |  |  |  |
| *CCND1* |  |  |  | 1 (NS) |
| Amplified | 13 (37%) | 5 (38%) | 8 (36%) |  |
| Wild type | 22 (63%) | 8 (62%) | 14 (64%) |  |
| *CDKN2A/B* |  |  |  | 0.1 (NS) |
| Co-deleted | 4 (11%) | 3 (23%) | 1 (5%) |  |
| Wild type | 31 (89%) | 10 (77%) | 21 (95%) |  |
| *CDKN2A* |  |  |  | 0.4 (NS) |
| Mutated | 6 (17%) | 1 (8%) | 5 (23%) |  |
| Wild type | 29 (83%) | 12 (92%) | 17 (77%) |  |
| *CDKN2A/B* |  |  |  | 1 (NS) |
| Altered^¶^ | 10 (29%) | 4 (31%) | 6 (27%) |  |
| Wild type | 25 (71%) | 9 (69%) | 16 (73%) |  |
| *EGFR* |  |  |  | 1 (NS) |
| Amplified | 3 (9%) | 1 (8%) | 2 (9%) |  |
| Wild type | 32 (91%) | 12 (92%) | 20 (91%) |  |
| *TERT* |  |  |  | 1 (NS) |
| Mutated | 8 (23%) | 3 (23%) | 5 (23%) |  |
| Wild type | 27 (77%) | 10 (77%) | 17 (77%) |  |
|  |  |  |  |  |
| Pathways |  |  |  |  |
| Wnt |  |  |  | 0.7 (NS) |
| Altered | 7 (20%) | 2 (15%) | 5 (23%) |  |
| Unaltered | 28 (80%) | 11 (85%) | 17 (77%) |  |
| Cell cycle |  |  |  | 0.8 (NS) |
| Altered | 19 (54%) | 8 (62%) | 11 (50%) |  |
| Unaltered | 16 (46%) | 5 (38%) | 11 (50%) |  |
| Senescence |  |  |  | 1 (NS) |
| Altered | 8 (23%) | 3 (23%) | 5 (23%) |  |
| Unaltered | 27 (77%) | 10 (77%) | 17 (77%) |  |
| Apoptosis |  |  |  | 0.7 (NS) |
| Altered | 8 (23%) | 2 (15%) | 6 (27%) |  |
| Unaltered | 27 (77%) | 11 (85%) | 16 (73%) |  |
| Chromatin organization | |  |  | 0.2 (NS) |
| Altered | 9 (26%) | 5 (38%) | 4 (18%) |  |
| Unaltered | 26 (74%) | 8 (62%) | 18 (82%) |  |
| Genome integrity |  |  |  | 1 (NS) |
| Altered | 23 (66%) | 9 (69%) | 14 (64%) |  |
| Unaltered | 12 (34%) | 4 (31%) | 8 (36%) |  |
| Hippo |  |  |  | 1 (NS) |
| Altered | 27 (77%) | 10 (77%) | 17 (77%) |  |
| Unaltered | 8 (23%) | 3 (23%) | 5 (23%) |  |
| RTK/RAS |  |  |  | 1 (NS) |
| Altered | 8 (23%) | 3 (23%) | 5 (23%) |  |
| Unaltered | 27 (77%) | 10 (77%) | 17 (77%) |  |
| Transcription factor regulator | |  |  | 1 (NS) |
| Altered | 5 (14%) | 2 (15%) | 3 (14%) |  |
| Unaltered | 30 (86%) | 11 (85%) | 19 (86%) |  |

Abbreviations: NS, not significant; ^¶^*CDKN2A/B* mutated or co-deleted
