# Supplementary material for: Randomized phase II study of preoperative afatinib in untreated head and neck cancers: predictive and pharmacodynamic biomarkers of activity
Source: Sci Rep. 2023 Dec 18;13:22524. doi: 10.1038/s41598-023-49887-4 (PMC10728082; doi:10.1038/s41598-023-49887-4)
Supplement: Supplementary file 21 — Supplementary Table 3. [file 41598_2023_49887_MOESM21_ESM.docx]

**Supplementary Table 3.** Prognostic value of genomic alterations and altered molecular pathways

|  | Patients  n (%) | PFS | *P*-value | OS | *P*-value |
| --- | --- | --- | --- | --- | --- |
|  |  | **Progression**  **n (%)** |  | **Death**  **n (%)** |  |
| Total | 56 (100%) | 24 (43%) |  | 21 (37.5%) |  |
| Genes |  |  |  |  |  |
| *CCND1* |  |  | **0.05** |  | 0.07 (NS) |
| Amplified | 19 (34%) | 11 (46%) |  | 10 (48%) |  |
| Wild type | 37 (66%) | 13 (54%) |  | 11 (52%) |  |
| *CDKN2A/B* |  |  | **0.02** |  | **0.004** |
| Co-deleted | 9 (16%) | 7 (29%) |  | 7 (33%) |  |
| Wild type | 47 (84%) | 17 (71%) |  | 14 (67%) |  |
| *CDKN2A* |  |  | 0.9 (NS) |  | 0.3 (NS) |
| Mutated | 12 (21%) | 5 (21%) |  | 3 (14%) |  |
| Wild type | 44 (79%) | 19 (79%) |  | 18 (86%) |  |
| *CDKN2A/B* |  |  | 0.1 (NS) |  |  |
| Altered^¶^ | 21 (38%) | 12 (50%) |  | 10 (48%) | 0.3 (NS) |
| Wild type | 35 (62%) | 12 (50%) |  | 11 (52%) |  |
| *EGFR* |  |  | 0.2 (NS) |  | 0.2 (NS) |
| Amplified | 3 (5%) | 0 |  | 0 |  |
| Wild type | 53 (95%) | 24 (100%) |  | 21 (100%) |  |
| *TERT* |  |  | 0.1 (NS) |  | 0.6 (NS) |
| Mutated | 13 (23%) | 3 (12%) |  | 4 (19%) |  |
| Wild type | 43 (77%) | 21 (88%) |  | 17 (81%) |  |
|  |  |  |  |  |  |
| Pathways |  |  |  |  |  |
| Wnt |  |  | 0.2 (NS) |  | 0.8 (NS) |
| Altered | 9 (16%) | 2 (8%) |  | 3 (14%) |  |
| Unaltered | 47 (84%) | 22 (92%) |  | 18 (86%) |  |
| Cell cycle |  |  | **0.03** |  | **0.01** |
| Altered | 33 (59%) | 18 (75%) |  | 17 (81%) |  |
| Unaltered | 23 (41%) | 6 (25%) |  | 4 (19%) |  |
| Senescence |  |  | 0.1 (NS) |  | 0.6 (NS) |
| Altered | 13 (23%) | 3 (12%) |  | 4 (19%) |  |
| Unaltered | 43 (77%) | 21 (88%) |  | 17 (81%) |  |
| Metabolism |  |  | 0.2 (NS) |  | 0.07 (NS) |
| Altered | 4 (7%) | 3 (12%) |  | 3 (14%) |  |
| Unaltered | 52 (93%) | 21 (88%) |  | 18 (86%) |  |
| Apoptosis |  |  | 0.6 (NS) |  | 0.6 (NS) |
| Altered | 12 (21%) | 7 (29%) |  | 4 (19%) |  |
| Unaltered | 44 (79%) | 17 (71%) |  | 17 (81%) |  |
| Chromatin organization | |  | 0.3 (NS) |  | 0.3 (NS) |
| Altered | 15 (27%) | 9 (38%) |  | 4 (19%) |  |
| Unaltered | 41 (73%) | 15 (62%) |  | 17 (81%) |  |
| Genome integrity |  |  | 0.7 (NS) |  | 0.9 (NS) |
| Altered | 35 (62%) | 14 (58%) |  | 13 (62%) |  |
| Unaltered | 21 (38%) | 10 (42%) |  | 8 (38%) |  |
| Hippo |  |  | 0.7 (NS) |  | 0.7 (NS) |
| Altered | 12 (21%) | 6 (25%) |  | 4 (19%) |  |
| Unaltered | 44 (79%) | 18 (75%) |  | 17 (81%) |  |
| PI3K |  |  | 0.9 (NS) |  | 0.9 (NS) |
| Altered | 7 (12%) | 3 (12%) |  | 3 (14%) |  |
| Unaltered | 49 (88%) | 21 (88%) |  | 18 (86%) |  |
| RTK/RAS |  |  | 0.7 (NS) |  | 0.9 (NS) |
| Altered | 9 (16%) | 4 (17%) |  | 3 (14%) |  |
| Unaltered | 47 (84%) | 20 (83%) |  | 18 (86%) |  |
| Transcription factor regulator | |  | 0.7 (NS) |  | 0.5 (NS) |
| Altered | 7 (12%) | 3 (12%) |  | 2 (10%) |  |
| Unaltered | 49 (88%) | 21 (88%) |  | 19 (90%) |  |

Abbreviations: PFS, progression-free survival; OS, overall survival; NS, not significant; ^¶^*CDKN2A/B* mutated or codeleted
