# Supplementary material for: Randomized phase II study of preoperative afatinib in untreated head and neck cancers: predictive and pharmacodynamic biomarkers of activity
Source: Sci Rep. 2023 Dec 18;13:22524. doi: 10.1038/s41598-023-49887-4 (PMC10728082; doi:10.1038/s41598-023-49887-4)
Supplement: Supplementary file 22 — Supplementary Table 4. [file 41598_2023_49887_MOESM22_ESM.docx]

**Supplementary Table 4.** Multivariate analyses of overall survival and progression-free survival

| Variables | Overall survival | | | Progression-free survival | | |
| --- | --- | --- | --- | --- | --- | --- |
|  | **HR** | **95% CI** | ***P*-value** | **HR** | **95% CI** | ***P*-value** |
| *CDKN2A/B* codeletion, yes (vs no) | 2.3 | 0.9-6.2 | 0.09 (NS) | 2.78 | 0.8-9.9 | 0.1 (NS) |
| *CCND1* amplification, yes (vs no) | 2.9 | 0.9-9.3 | 0.07 (NS) | 2.41 | 0.7-8.1 | 0.2 (NS) |
| Sex, male (vs female) | 0.8 | 0.3-2.2 | 0.7 (NS) | 0.8 | 0.3-2.2 | 0.7 (NS) |
| Age (years) | 1 | 0.9-1.1 | 1 (NS) | 1.02 | 1-1.1 | 0.4 (NS) |
| Clinical T-stage, T4 (vs T1/T2/T3) | 1.8 | 0.7-4.7 | 0.2 (NS) | 1.9 | 0.8-4.8 | 0.2 (NS) |
| Tumor location |  |  |  |  |  |  |
| Larynx vs hypopharynx | 1.6 | 0.1-24 | 0.7 (NS) | 1.6 | 0.1-24 | 0.7 (NS) |
| Oral cavity vs hypopharynx | 0.8 | 0.2-35 | 0.8 (NS) | 0.8 | 0.2-35 | 0.8 (NS) |
| Oropharynx vs hypopharynx | 0.4 | 0.07-2.7 | 0.4 (NS) | 0.4 | 0.07-2.7 | 0.4 (NS) |

Abbreviations: CI, confidence interval; HR, hazard ratio; NS, not significant
