# Supplementary material for: Randomized phase II study of preoperative afatinib in untreated head and neck cancers: predictive and pharmacodynamic biomarkers of activity
Source: Sci Rep. 2023 Dec 18;13:22524. doi: 10.1038/s41598-023-49887-4 (PMC10728082; doi:10.1038/s41598-023-49887-4)
Supplement: Supplementary file 23 — Supplementary Table 5. [file 41598_2023_49887_MOESM23_ESM.docx]

**Supplementary Table 5.** Most differentially expressed B cell-related genes under afatinib, by

metabolic response according to PERCIST

| **Genes** | **Non-responders**  **(*N*=11)** | | | | **Responders**  **(*N*=15)** | | | **Ratio of median** | | ***P*-value** |
| --- | --- | --- | --- | --- | --- | --- | --- | --- | --- | --- |
|  | Median^$^ | Min | Max | Median^$^ | | Min | Max | |  |  |
| ***ICAM1*** | 1.4 | 0.4 | 13.1 | 0.6 | | 0.2 | 2.9 | | 0.4 | **0.04** |
| ***MEF2C*** | 1.2 | 0.2 | 4.7 | 2.3 | | 0.6 | 20.3 | | 1.9 | **0.04** |
| ***P2RX5*** | 1 | 0.02 | 2.3 | 2.2 | | 0.2 | 224.1 | | 2.2 | **0.007** |
| ***HPGD*** | 0.5 | 0.2 | 4.2 | 3.2 | | 0.3 | 170.2 | | 6.2 | **0.03** |
| ***ZBTB16*** | 3.2 | 0.01 | 28.4 | 48.1 | | 0.7 | 376.1 | | 15 | **0.0004** |

^$^Median post-afatinib/pre-afatinib ratios of gene expression levels
