# Supplementary material for: Randomized phase II study of preoperative afatinib in untreated head and neck cancers: predictive and pharmacodynamic biomarkers of activity
Source: Sci Rep. 2023 Dec 18;13:22524. doi: 10.1038/s41598-023-49887-4 (PMC10728082; doi:10.1038/s41598-023-49887-4)
Supplement: Supplementary file 24 — Supplementary Table 6. [file 41598_2023_49887_MOESM24_ESM.docx]

**Supplementary Table 6.** Characteristics of patients in the two clusters identified by unsupervised analysis on bulk gene expression data in 53 baseline biopsies

|  | Cluster 1  (*N*=27) | Cluster 2  (*N*=26) | *P*-value |
| --- | --- | --- | --- |
| **Age, years** |  |  |  |
| Mean (SD) | 58.7 (8.6) | 59.1 (8.1) | 0.9 |
| **Sex, n (%)** |  |  |  |
| Female | 5 (18%) | 6 (23%) | 0.9 |
| Male | 22 (82%) | 20 (77%) |  |
| **Smoker, n (%)** |  |  | 1 |
| Current | 15 (56%) | 14 (54%) |  |
| Never/Former | 12 (44%) | 2 (46%) |  |
| **Alcohol consumption, n (%)** |  |  |  |
| Current | 12 (44%) | 15 (58%) | 0.5 |
| Never/former | 15 (56%) | 11 (42%) |  |
| **HPV status, n (%)** |  |  | **0.04** |
| Negative | 26 (96%) | 18 (75%) |  |
| Positive | 1 (4%) | 6 (25%) |  |
| **T classification, n (%)** |  |  | 1 |
| T1/T2 | 13 (68%) | 13 (50%) |  |
| T3/T4 | 14 (52%) | 13 (50%) |  |
| **Grading, n (%)** |  |  | 0.3 |
| Poorly/Moderately differentiated | 9 (36%) | 14 (54%) |  |
| Well differentiated | 16 (64%) | 12 (46%) |  |
| **Tumour location, n (%)** |  |  | 0.7 |
| Hypopharynx/Oropharynx/Larynx | 7 (26%) | 9 (35%) |  |
| Oral cavity | 20 (74%) | 17 (65%) |  |
| **Lymph node involvment, n (%)** |  |  | 0.9 |
| Negative | 15 (56%) | 13 (50%) |  |
| Positive | 12 (44%) | 13 (50%) |  |

Abbreviation: SD, standard deviation
