# Supplementary material for: Randomized phase II study of preoperative afatinib in untreated head and neck cancers: predictive and pharmacodynamic biomarkers of activity
Source: Sci Rep. 2023 Dec 18;13:22524. doi: 10.1038/s41598-023-49887-4 (PMC10728082; doi:10.1038/s41598-023-49887-4)
Supplement: Supplementary file 25 — Supplementary Table 7. [file 41598_2023_49887_MOESM25_ESM.docx]

**Supplementary Table 7.** Pathways and genes analysed

in the GEP 11 PREDICTOR trial

| **Pathways** | **Genes** |
| --- | --- |
| **Genome integrity** | *TP73*  *TP53* |
| **Cell cycle** | *CDKN2A*  *RB1*  *CCND1*  *CCND2*  *CCND3*  *CDKN1A*  *CDKN1B*  *CDK4*  *CDK6*  *CDKN2B*  *CDKN2C*  *CCNE1*  *SLX4*  *CUL4B*  *MBD4*  *NTHL1*  *POLR2D*  *SMUG1*  *TDG*  *WRN*  *MDM2*  *MDM4*  *BRCC3*  *RNF169*  *AURKA*  *STAG1*  *STAG2*  *SMC1A*  *SMC3* |
| **Chromatin organization** | *ACTL6A*  *ACTL6B*  *KDM6A*  *MECOM*  *SMARCE1*  *TRRAP*  *KDM5C*  *BRD7*  *GAS8-AS1*  *PBRM1*  *BMI1*  *DPF1*  *DPF2*  *DPF3*  *EMSY*  *H3F3A*  *H3F3B*  *HIST1H3B*  *HIST1H1B*  *HIST1H3C*  *HIST2H3C*  *ASXL1*  *ASXL2*  *SETBP1* |
| **Senescence** | *TERT*  *POT1*  *TERC* |
| **Apoptosis** | *CASP8*  *ING1*  *BCL7C*  *SAMD9*  *BCL11B*  *APAF1*  *BCL2*  *BCL2L1*  *BIRC2*  *BIRC3*  *DAXX*  *FADD*  *FAS*  *MCL1*  *CDC25C*  *CDC27*  *NIPBL*  *NUMA1*  *PRIM2*  *RBBP6*  *PPP6C*  *BTG1* |
| **Hippo** | *NF2*  *AJUBA*  *FAT1*  *FAT2*  *FAT4* |
| **Receptor tyrosine kinase/RAS** | *BRAF*  *ERBB2*  *ERBB3*  *ERBB4*  *HRAS*  *KRAS*  *MAP2K1*  *MAP2K2*  *MAP2K4*  *MAP2K7*  *MAP3K1*  *MAPK1*  *NRAS*  *PDGFRA*  *AXL*  *CBLC*  *CXCR4*  *EPHA3*  *EPHA7*  *EPHA5*  *EPHB1*  *EPHB2*  *GNA11*  *MST1R*  *PLCB4*  *RIT1*  *AGTR2*  *ALK*  *DDR2*  *PDGFRB*  *RASA1*  *RASA2*  *EGFR*  *FGF19*  *FGF3*  *FGF4*  *FGFR4*  *IGF1R*  *FGF10*  *FGF6*  *FGFR1*  *FGFR2*  *FGFR3*  *VEGFA*  *RAC1*  *LCK*  *LYN*  *BTK*  *GNAQ*  *GNAS*  *KIT*  *MET*  *RET*  *ROS1*  *IGF2R*  *RHOA*  *EPHA2* |
| **Wnt** | *CSNK1A1*  *AMER1*  *RNF43*  *APC2*  *APC*  *AXIN1*  *AXIN2*  *TCF7L2*  *CTNNA1*  *CTNNB1* |
| **PI3K** | *PIK3CA*  *PIK3CB*  *PIK3CG*  *PIK3R1*  *PIK3R2*  *PTEN*  *PPP2R1A*  *AKT1*  *AKT2*  *AKT3*  *MTOR*  *RHEB*  *RPTOR*  *STK11*  *TSC1*  *TSC2*  *RICTOR*  *PREX2*  *INPP4B*  *VHL* |
| **Transcription factor-regulator** | *AR*  *BCL11A*  *BCL6*  *CBFB*  *CDK8*  *CEBPA*  *CITED2*  *DDX3X*  *DDX41*  *EBF1*  *ELF3*  *ESR1*  *ETV6*  *FOXA1*  *FOXL2*  *FOXO1*  *FOXP1*  *FOXR2*  *GFI1*  *HOXB13*  *ID3*  *IKZF1*  *IKZF3*  *KLF2*  *KLF4*  *CTCF*  *GATA1*  *GATA2*  *GATA3*  *GATA6*  *HNF1A*  *NCOR1*  *NCOR2*  *PHOX2B*  *RUNX1*  *ZFHX3*  *ZFP36L1*  *ZFP36L2*  *PGR*  *BCOR*  *BCORL1* |
| **DNA repair** | *BARD1*  *BRCA1*  *BRCA2*  *FANCC*  *MLH1*  *MSH2*  *MSH3*  *MSH6*  *POLD1*  *POLE*  *POLE2*  *RAD50*  *RAD51*  *RAD51B*  *KANSL1*  *CHD1*  *CHD2*  *CHD3*  *CHD4*  *CHD6*  *CHD8*  *CHD9*  *ATM*  *CHEK1*  *CHEK2*  *RAD51C*  *RAD51D*  *BRIP1*  *FANCA*  *MUTYH*  *CDK12*  *ATR*  *BLM*  *FANCD2*  *FANCE*  *FANCF*  *FANCG*  *FANCI*  *FANCL*  *FANCM*  *MRE11*  *NBN*  *PALB2*  *PMS2*  *XRCC1*  *XRCC2*  *XRCC3*  *RAD54L*  *ERCC2*  *FANCB*  *RECQL4*  *MLH3*  *RAD21*  *BAP1*  *ATRX*  *PMS1*  *TP53*  *BP1* |
| **Cellular metabolism** | *ABHD5*  *CTPS1*  *CYP1A1*  *CYP2D6*  *ABCB1*  *ABCG2*  *DCK*  *DCTD*  *DPYD*  *ETNK1*  *EXT1*  *EXT2*  *FH*  *ITPKB*  *LDLRAP1*  *LEPROTL1*  *MTHFR*  *NQO1*  *NT5C2*  *SDHD*  *SLC1A2*  *SLC29A1*  *SLC2A9*  *SNCAIP*  *SNX25*  *IDH2*  *IDH1*  *SDHA*  *SDHB*  *SDHC*  *PHGDH* |
| **Immunity** | *B2M*  *BCL10*  *CIITA*  *CARD11*  *CBL*  *CBLB*  *CD274*  *CD28*  *CD36*  *CD58*  *CD70*  *CD79A*  *CD79B*  *CSF1R*  *CSF3R*  *ELANE*  *HLA-A*  *HLA-B*  *HLA-C*  *IRF2*  *IRF4*  *IRF8*  *MALT1*  *MYD88*  *NFKBIE*  *TLR4*  *TNF*  *TNFAIP3*  *TNFRSF14*  *TRAF2*  *TRAF3*  *WAS*  *WASF1*  *WASF2*  *WASF3*  *KLHL6*  *MFHAS1* |
| **JAK-STAT** | *JAK1*  *JAK2*  *JAK3*  *MPL*  *STAT3*  *STAT5B*  *STAT6*  *SOCS1*  *SH2B3*  *ZDHHC19*  *IL6ST* |
| **TGF-beta** | *SMAD2*  *SMAD3*  *SMAD4*  *TGFBR2*  *ACVR1*  *ACVR1B*  *ACVR2A*  *ACVR2B*  *BMPR2* |
| **Other** | *NCKAP1*  *NCKAP1L*  *PTPRB*  *THSD7B*  *ALPK2*  *CIC*  *U2AF1*  *U2AF2*  *NUP214*  *NUP93*  *PIM1*  *PLCG2*  *PRKCI*  *PRKD1*  *PRKD2*  *RIMS1*  *SGK1*  *AKAP9*  *XPO1*  *FRS2*  *IRS2*  *ALDOA*  *BRK1*  *CDA*  *THBS1*  *ABL1*  *GSTP1*  *UBE2K*  *ADGRB3*  *ANKRD26*  *BC040327*  *CAND1.11*  *CCDC107*  *CRBN*  *FAM213A*  *FRG1BP*  *FSHR*  *LINC02008*  *LOC101927630*  *MYO3A*  *NAV3*  *PLEKHS1*  *SAMD9L*  *SARAF*  *STMN2*  *TBC1D12*  *ADGRG6*  *CUX1*  *FBXO11*  *FUBP1*  *GRIN2A*  *LZTR1*  *PXDNL*  *SPOP*  *SRCAP*  *TBL1XR1*  *ZRSR2*  *GNA13* |
