# Supplementary material for: Randomized phase II study of preoperative afatinib in untreated head and neck cancers: predictive and pharmacodynamic biomarkers of activity
Source: Sci Rep. 2023 Dec 18;13:22524. doi: 10.1038/s41598-023-49887-4 (PMC10728082; doi:10.1038/s41598-023-49887-4)
Supplement: Supplementary file 26 — Supplementary Table 8. [file 41598_2023_49887_MOESM26_ESM.docx]

**Supplementary Table 8.** Proteins and primary antibodies investigated by RPPA

| **Protein/Antigen** | **Supplier link** | **Oncogenic pathway** |
| --- | --- | --- |
| Phospho-Rb (Ser807/811) | ['9308'](http://www.cellsignal.com/products/9308.html) | Cell cycle |
| Ki67 (MIB-1) | ['M7240'](http://www.dako.com/fr/searchresultlist?search=M7240&submit=Search) | Cell cycle |
| Rb | ['9309 (4H1)'](http://www.cellsignal.com/products/9309.html) | Cell cycle |
| Vimentin (D21H3) | ['#5741'](http://www.cellsignal.com/products/#5741.html) | Cell migration |
| FAK | ['3285'](http://www.cellsignal.com/products/3285.html) | Cell migration |
| Phospho-FAK (Tyr861) | ['ab81293 (epitomics 2153-1)'](http://www.abcam.com/ab81293%20(epitomics%202153-1)) | Cell migration |
| Cleaved PARP (Asp214) p25 | ['ab32064'](http://www.epitomics.com/index.php/products/search/ab32064) | Genome integrity |
| Rad50 | ['3427'](http://www.cellsignal.com/products/3427.html) | Genome integrity |
| Phospho-NDRG1 (Thr346) | ['5482'](http://www.cellsignal.com/products/5482.html) | Genome integrity |
| NDRG1 | ['5100-1'](http://www.epitomics.com/index.php/products/search/5100-1) | Genome integrity |
| Phospho-ATM (ser1981) | ['NB110-55475'](http://www.novusbio.com/productsearch/NB110-55475) | Genome integrity |
| Rad51 (D4B10) | ['8875'](http://www.cellsignal.com/products/8875.html) | Genome integrity |
| ATM | ['1549-1'](http://www.epitomics.com/index.php/products/search/1549-1) | Genome integrity |
| Phospho-Histone H2AX (ser139) | ['ab2893'](http://www.abcam.com/ab2893) | Genome integrity |
| p53 | ['9282'](http://www.cellsignal.com/products/9282.html) | Genome integrity |
| Phospho-Topoisomerase II a (Thr1343) | ['1871-1 / ab52853'](http://www.epitomics.com/index.php/products/search/1871-1%20/%20ab52853) | Genome integrity |
| Histone H2AX | ['2595'](http://www.cellsignal.com/products/2595.html) | Genome integrity |
| Phospho-Chk2 (thr68) (C13C1) | ['2197'](http://www.cellsignal.com/products/2197.html) | Genome integrity |
| B7-H4 | ['14572'](http://www.cellsignal.com/products/14572.html) | Immunity |
| IDO (D5J4E) | ['#86630'](http://www.cellsignal.com/products/#86630.html) | Immunity |
| PD-L1 (E1L3N) | ['13684'](http://www.cellsignal.com/products/13684.html) | Immunity |
| Phospho-PTP1B (Ser378) | ['2163-1'](http://www.epitomics.com/index.php/products/search/2163-1) | JAK/STAT |
| PTP1B | ['2066-1'](http://www.epitomics.com/index.php/products/search/2066-1) | JAK/STAT |
| Stat3 | ['4904'](http://www.cellsignal.com/products/4904.html) | JAK/STAT |
| Phospho-Stat1 (Y701) | ['ab109457'](http://www.abcam.com/ab109457) | JAK/STAT |
| Phospho-Stat3 (Ser727) | ['9134'](http://www.cellsignal.com/products/9134.html) | JAK/STAT |
| Stat1 | ['9172'](http://www.cellsignal.com/products/9172.html) | JAK/STAT |
| Phospho-p38 MAPK (Thr180/Tyr182) | ['4631'](http://www.cellsignal.com/products/4631.html) | MAPK |
| p38 MAPK | ['1544-1'](http://www.epitomics.com/index.php/products/search/1544-1) | MAPK |
| MEK1/2 | ['9122S'](http://www.cellsignal.com/products/9122S.html) | MAPK |
| p44/42 MAPK | ['9102'](http://www.cellsignal.com/products/9102.html) | MAPK |
| Phospho-MEK1/2 (Ser217/221) | ['9154'](http://www.cellsignal.com/products/9154.html) | MAPK |
| Phospho-p44/42 MAPK (Thr202/Tyr204) | ['4377 (197G2)'](http://www.cellsignal.com/products/4377.html) | MAPK |
| AMPK alpha | ['2532'](http://www.cellsignal.com/products/2532.html) | Metabolism |
| Phospho-AMPK beta1 (Ser181) | ['2271-1'](http://www.epitomics.com/index.php/products/search/2271-1) | Metabolism |
| PKM2 | ['4053S'](http://www.cellsignal.com/products/4053S.html) | Metabolism |
| CA9 (D47G3) | ['5649S'](http://www.cellsignal.com/products/5649S.html) | Metabolism |
| LKB1 | ['3050'](http://www.cellsignal.com/products/3050.html) | Metabolism |
| phospho-PKM2(Tyr105) | ['3827P'](http://www.cellsignal.com/products/3827P.html) | Metabolism |
| IKK beta | ['2370'](http://www.cellsignal.com/products/2370.html) | NF-kB |
| NF-kB p65 | ['4764 (C22B4)'](http://www.cellsignal.com/products/4764.html) | NF-kB |
| RelB | ['1653-1'](http://www.epitomics.com/index.php/products/search/1653-1) | NF-kB |
| Phospho-NF-kB p65 (Ser536) | ['3033'](http://www.cellsignal.com/products/3033.html) | NF-kB |
| Phospho-S6 Ribosomal (Ser240/244) | ['2215'](http://www.cellsignal.com/products/2215.html) | PI3K |
| PTEN (D4.3) XP | ['9188S'](http://www.cellsignal.com/products/9188S.html) | PI3K |
| S6 Ribosomal Protein (5G10) | ['2217'](http://www.cellsignal.com/products/2217.html) | PI3K |
| Phospho-Akt (Ser473) (193H12) | ['4058'](http://www.cellsignal.com/products/4058.html) | PI3K |
| Phospho-S6 Ribosomal (Ser235/236) | ['2211'](http://www.cellsignal.com/products/2211.html) | PI3K |
| Phospho-Akt (Thr308) (D25E6) | ['13038'](http://www.cellsignal.com/products/13038.html) | PI3K |
| Phospho-PTEN (ser380/Thr382/383) | ['9554'](http://www.cellsignal.com/products/9554.html) | PI3K |
| Akt | ['9272'](http://www.cellsignal.com/products/9272.html) | PI3K |
| Phospho-HER4 (Tyr1162) | ['2295-1'](http://www.epitomics.com/index.php/products/search/2295-1) | RTK |
| Phospho-Her3/Erbb3 (tyr1289) | ['4791'](http://www.cellsignal.com/products/4791.html) | RTK |
| HER3/ErbB3 (c-17) | ['sc-285'](http://www.tebu-bio.com/file/product/036sc-285) | RTK |
| Phospho-EGFR (Tyr1068) (D7A5) | ['3777'](http://www.cellsignal.com/products/3777.html) | RTK |
| EGFR (D38B1) | ['4267'](http://www.cellsignal.com/products/4267.html) | RTK |
| Met (D1C2) XP | ['8198'](http://www.cellsignal.com/products/8198.html) | RTK |
| HER4/ErbB4 | ['76303 (2218-1/Epitomics)'](http://www.abcam.com/76303%20(2218-1/Epitomics)) | RTK |
| Phospho-HER2/ErbB2 (Tyr1139) | ['1991-1; ab53290'](http://www.abcam.com/1991-1;%20ab53290) | RTK |
| HER2/ErbB2 | ['MA5-14057'](https://www.thermofisher.com/order/genome-database/antibody/HER-2-ErbB2-Antibody-clone-e2-4001-3B5-Monoclonal/MA5-14057) | RTK |
| Phospho-Met (Tyr1234/1235) | ['3129'](http://www.cellsignal.com/products/3129.html) | RTK |
| IGF-I Receptor beta | ['3027'](http://www.cellsignal.com/products/3027.html) | RTK |
| Phospho-EGFR (Tyr 1173) (53A5) | ['4407'](http://www.cellsignal.com/products/4407.html) | RTK |
| Phospho-p70 S6 Kinase (Thr389) | ['9205'](http://www.cellsignal.com/products/9205.html) | TOR |
| mTOR | ['ab51089'](http://www.abcam.com/ab51089) | TOR |
| Phospho-p70 S6 kinase (Thr421/Ser424) | ['04-393'](http://www.millipore.com/catalogue/item/04-393) | TOR |
| phospho-mTOR (Ser2448) | ['ab109268'](http://www.abcam.com/ab109268) | TOR |
| p70 S6 Kinase | ['2708'](http://www.cellsignal.com/products/2708.html) | TOR |
| E-cadherin | ['610181'](http://www.bdbiosciences.com/ecat/Searchresults.do?pgNum=1&pgSize=&sort=SortOrderDef&check=mainsearchcheck&key=610181&mterms=true) | Wnt/beta-catenin |
| Phospho-YAP65 (Ser127) | ['4911S'](http://www.cellsignal.com/products/4911S.html) | Wnt/beta-catenin |
| Cleaved Notch1 (Val1744) (D3B8) | ['4147'](http://www.cellsignal.com/products/4147.html) | Wnt/beta-catenin |
| Notch1 (D1E11) | ['3608'](http://www.cellsignal.com/products/3608.html) | Wnt/beta-catenin |
| YAP65 | ['2060-1'](http://www.epitomics.com/index.php/products/search/2060-1) | Wnt/beta-catenin |
| Phospho-Beta Catenin (Ser675) | ['4176'](http://www.cellsignal.com/products/4176.html) | Wnt/beta-catenin |
| Phospho-Beta Catenin (Ser552) (D8E11) | ['5651'](http://www.cellsignal.com/products/5651.html) | Wnt/beta-catenin |
| JunB | ['3753'](http://www.cellsignal.com/products/3753.html) | Wnt/beta-catenin |
| Beta Catenin (6B3) | ['9582'](http://www.cellsignal.com/products/9582.html) | Wnt/beta-catenin |
